# Partisan Animosity as Blame: A Unifying and Generative Framework for Understanding and Transforming Affective Polarization in the Political Sphere

**DOI:** 10.1177/10888683251407825

**Published:** 2026-01-03

**Authors:** Raihan Alam, Michael Gill

**Affiliations:** 1 University of California San Diego, USA; 2 Lehigh University, Bethlehem, PA, USA

**Keywords:** partisan animosity, blame, moral psychology, interventions

## Abstract

**Academic Abstract:**

Partisan animosity is on the rise in many nations around the globe. Given its significant implications, it is imperative to establish a clear conceptualization of partisan animosity that can aid efforts to reduce it. To address this need, we present a novel framework that conceptualizes partisan animosity as an attitude of blame directed toward political outgroups. Drawing from the literature on moral psychology, we construct a comprehensive model of the psychology of blame. Then, we use that model as an interpretive lens to understand existing interventions that have reduced partisan animosity. Finally, we suggest a variety of possible future interventions inspired by our framework. By adopting this blame-based perspective, our article sheds light on the underlying mechanisms of partisan animosity, provides a unifying framework for understanding existing work, and stimulates novel ideas for future research.

**Public Abstract:**

Partisan animosity, hostility directed toward political outparties, has been growing in many areas of the world, with significant negative impacts on society and politics. This article offers a new perspective on this growing animosity, proposing that partisan animosity reflects an attitude of blame that partisans direct toward each other. Drawing from insights in moral psychology, we present a model of blame, describing how it operates, and use the model to understand both the nature of partisan animosity and potential pathways for intervention. Our model contributes to understanding partisan animosity with the ultimate goal of informing interventions to reduce it.

Nations around the globe are witnessing an alarming rise in partisan animosity—hostile thoughts, feelings, and behaviors directed toward a political outgroup (Hartman et al., 2022; Voelkel et al., 2023). In the United States, the extent to which Americans hold cold feelings toward the political outgroup is at the highest it has been in the last four decades (Finkel et al., 2020; Iyengar et al., 2019). Indeed, most Democrats and Republicans view members of the outparty as immoral, dishonest, unintelligent, and closed-minded (Nadeem, 2022). This pattern is not unique to the United States. Comparative analyses of Western democracies reveal that hostility toward outparties is similarly high in countries such as Australia, Canada, France, Israel, Spain, and Switzerland (Gidron et al., 2019).

Such high levels of animus do not come without consequences. Some of these consequences are political: Partisan animosity has been associated with being unwilling to compromise with political outparty members (Levendusky, 2018), and with only trusting the government when the inparty is in power (Hetherington, 2015). A lack of trust in the outparty can contribute to self-harming behaviors, such as ignoring health guidelines during the COVID-19 pandemic (Van Bavel et al., 2024), and to other-harming behaviors, such as keeping schools closed longer than necessary during the pandemic (Leonhardt, 2024), which particularly impacted vulnerable children (Betthäuser et al., 2023). Partisan animosity has even been linked to endorsing violence against outparty members (Kalmoe & Mason, 2022), and we have seen many recent instances of such violence (Jacobson & Sherman, 2025). Other negative consequences of partisan animosity show up in everyday interpersonal contexts, with partisans rejecting outparty members as potential dates (Huber & Malhotra, 2017), employees (Gift & Gift, 2015), and scholarship winners (Iyengar & Westwood, 2015). Partisans even spend less time at holiday gatherings when visiting family members from the outparty (Chen & Rohla, 2018)!

Beyond examining its causes and consequences, scholars have expended considerable effort studying interventions to reduce partisan animosity (Hartman et al., 2022; Voelkel et al., 2024). We are among those scholars. Unlike most other scholars tackling this issue, however, our work grows out of an interest in the psychology of blame. Other work on depolarization has been rooted in alternative frameworks, including intergroup contact theory, social identity theory, and correcting misperceptions (see Hartman et al., 2022; Voelkel et al., 2024). Perhaps unsurprisingly, given our theoretical background, we view partisan animosity as an instance of blame. Furthermore, we contend that insights from the blame literature can shed light on the dynamics of partisan animosity, offering valuable insights to reduce it. Our intended contribution in this article is to connect the rich literature on the psychology of blame to the interdisciplinary literature on the pressing societal problem of partisan animosity. The literature on blame offers an abundant source of mechanisms to consider when one wishes to tackle the difficult problem of tempering mutual moralized disdain between ideological groups.

Below, we will first argue that partisan animosity can be effectively conceptualized as a manifestation of an attitude of blame. Then, we will show how existing theories regarding the roots and accelerants of partisan animosity are compatible with a blame-centric conceptualization. Third, we will introduce a general model of the psychology of blame derived from existing literature, a literature that generally does not examine the political realm. We suggest that the forces that increase or decrease partisan animosity do so by altering the determinants of one’s attitude of blame. Then, we will show that this model provides a unifying framework for understanding much of the existing work on partisan animosity. This will be done by classifying current interventions as addressing factors related to blame mitigation. Finally, we will conclude by offering ideas for future interventions inspired by the notion of partisan animosity as an attitude of blame toward an outparty.

## Ideological Polarization versus Partisan Animosity

Prior to delving into our analysis of partisan animosity as blame, we must distinguish partisan animosity from other related concepts. For example, many scholars study *ideological polarization* or *issue polarization* (Fiorina & Abrams, 2008), which is distinguished from *affective polarization* or *partisan animosity* (Iyengar et al., 2012). *Ideological polarization* refers to the extent to which political parties within a society disagree over political issues and possible solutions to societal problems (e.g., *Democrats think we should increase welfare payments, Republicans think we should strive to create more job opportunities*), whereas *partisan animosity* refers to the extent to which members of one party despise members of the other party (i.e., *Democrats and Republicans hate each other*). Although ideological polarization can sometimes contribute to partisan animosity (Bougher, 2017), in general, the two are only moderately related (Iyengar et al., 2012; Mason, 2015). One reason for this is that groups can disagree about the best solutions to social problems without hating each other. Another important reason to distinguish ideological polarization from partisan animosity is that ideological polarization—if not connected to animosity—can be healthy for a democracy, as it can expose individuals to diverse perspectives, which can help them generate better solutions to social problems (e.g., Shi et al., 2019). In contrast, we have not come across any evidence suggesting the benefits of mutual hatred.

Our introductory paragraphs highlight an important reason for focusing on partisan animosity rather than ideological polarization: Animosity is more dangerous to democracy and to societal health. Another important reason for our focus is that partisan animosity is on the rise in many areas of the world, including in Canada (Boxell et al., 2024), European countries such as Denmark, France, Switzerland, Poland, and Portugal (Boxell et al., 2024; Garzia et al., 2023; Reiljan, 2020), Oceanic countries such as New Zealand (Boxell et al., 2024; Garzia et al., 2023), Latin American countries including Brazil and Mexico (Bergman & Fernández, 2025), Middle Eastern countries such as Israel (Amitai et al., 2023), and in Asian countries like South Korea and Taiwan (Hsiao & Yu, 2025). Although its rise is steepest in the United States (Boxell et al., 2024), some research shows that current levels in the United States are not a global outlier compared to other democracies (Gidron et al., 2019). Therefore, our work may be useful in many areas around the globe.

## Partisan Animosity as Blame

The storm of partisan hostility currently battering the integrity of political institutions around the globe includes heated accusations of outparty depravity intertwined with angry, condemning emotions directed toward the outparty. In other words, partisan animosity is a manifestation of blame. To make this point concrete, we note that the following should sound all-too-familiar to our readers (although, naturally, the specific accusations will differ by nation):

Disgusting! Shameful! Disgraceful! Outrageous! Democrats want to steal money from hardworking Americans and give it away to lazy good-for-nothings! Republicans are callous monsters who want to take away healthcare from needy children so billionaires can get even richer! Liberals are out to destroy our precious inheritance of the values of Western Civilization! Conservatives are racists and xenophobes who want to keep White Christians in power forever! My liberal uncle wants to get rid of the brave police officers who keep us safe! My conservative uncle says that Black people who get beat up by the police probably deserved it! Democrats rigged the 2020 election! Republicans are spreading COVID misinformation that will harm millions! The Left turned [Black victims] Trayvon Martin and George Floyd into sacred martyrs but cannot muster any compassion for [White victims] Tony Timpa and Daniel Shaver! Republicans want Charlie Kirk to be a martyr, but he spewed hatred and ultimately got what was coming to him! Liberals don’t believe in the values that made our country great! Trump and his MAGA mob are determined to destroy American Democracy! That popular conversative pundit said that transgender people are deranged and demonic! That popular liberal pundit said that conservatives are a bunch of illiterate rednecks! Democrats are begging immigrants to invade and undermine our country! Republicans think immigrants should be treated as less-than-human and sent to rot in horrendous foreign prisons!

### The Nature of Blame

What does it mean to say that partisan animosity is a manifestation of blame? In part, it means that partisan animosity takes the form of the statements in the preceding paragraph: Hostility toward the outparty driven by perceptions of their supposedly profound immorality. To provide a deeper answer to this question, we must start by clarifying what we mean by blame. This is important because our conceptualization of blame differs from some other views in social psychology. Our conceptualization is informed by philosophical work on the nature of blame (see Coates & Tognazzini, 2013 for an overview). Like moral philosophers, we are not interested in the form of blame that simply assigns causality (*I blame the drought for killing my grass*.). Instead, we are interested in blame as *an attitude one takes toward wrongdoers*. More specifically, we characterize blame as *a hostile attitude targeting individuals or groups who are perceived as thinking or behaving in immoral ways, as thinking or doing “wrong” or “bad” things* (Gill & Cerce, 2021). An attitude of blame can range from mild (*I’m not a fan of the outparty because I find some of their beliefs slightly offensive*) to intense (*I hate the outparty because their beliefs are disgusting and evil*). Rising affective polarization is, in our view, an increase in the intensity of the attitude of blame each party takes toward the other(s).

One aspect that distinguishes our conceptualization most sharply from others in social psychology is our focus on hostile emotions being a typical feature of an attitude of blame. That is, an attitude of blame includes *other-condemning emotions* (Haidt, 2003), such as feelings of annoyance, irritation, anger, disgust, or hatred. Also integral to blame are cognitive and behavioral intention components. Cognitively, an attitude of blame involves derogatory thoughts regarding the traits of the blamed target, such as perceiving them as globally “bad,” or, in terms of more specific bad traits, such as callous, thoughtless, unfair, or even evil. With respect to behavior, blame involves behavioral intentions that reflect a goal of transforming offenders into better people (Funk et al., 2014) or, at least, preventing them from engaging in bad acts (Malle et al., 2014). Certainly, efforts to transform or regulate a blamed target can involve hostile behavioral intentions, such as the desire to “give them a piece of my mind,” “own them,” enact revenge, or otherwise subject the blamed target to unkind treatment (e.g., Gill & Cerce, 2017). Importantly, however, an attitude of blame can contribute to more constructive behavioral intentions—such as a desire to transform the transgressor via respectful persuasion—when the hostile affect of the blame attitude is tempered (Gill & Cerce, 2017, 2021).

Whereas affect plays a central role in our conceptualization, other theorists emphasize a more purely cognitive conceptualization of blame (Malle et al., 2014). Cognitive accounts conceptualize blame as a relatively “cool” *judgment*, as opposed to a “hot,” *hostile attitude*. Before contrasting these accounts further, we must point out what they have in common. All accounts agree that the process of blaming begins with perceived immorality connected to some target person or group, which is followed by cognitive appraisals centered on assessing the *blameworthiness* of the target for that immorality (Gill & Cerce, 2021; Malle, 2021; Malle et al., 2014). All accounts further agree that the cognitive appraisals relevant to assessing blameworthiness center on the target’s intentionality and reasons for acting, justification or lack thereof, degree of free will, and so on (Gill & Cerce, 2017; Malle et al., 2014). This is a lot of common ground. Where the accounts differ, however, is in their conceptualization of blame *per se*. That is, they differ in how they conceptualize what it is, precisely, that is triggered by the appraisal that a target is blameworthy. Cognitive accounts assert that it is a judgment: *In light of all the information I have, I make the decision to blame him.* Our account asserts that it is an affect-laden attitude: *In light of all the information I have, I hate him and I think he’s a complete jerk!*

As should be clear, we believe that blame is best conceptualized as an affect-laden attitude. We agree with the criticism that purely cognitive accounts of blame “leave the blame out of blame” (Wallace, 2011) by portraying blame as a detached, fact-finding assessment. Such portrayals fail to capture the *reactive attitudes* (*I resent her for treating me badly; I’m pissed at him for his selfishness*; Strawson, 1963) that seem integral to blame in daily life (Coates & Tognazzini, 2013). McGeer (2013), for instance, argues that cognitive accounts “sanitize” blame, failing to capture the “ordinary connotations of the term” which include “resentment and anger and the desire for payback” (p. 163). She further argues that “blame is typically emotionally toned, manifested in a variety of expressive behaviors that will often be experienced *as* punitive by those to whom it’s directed” (italics in original; p. 166). This affect-centric view is “widely accepted [among moral philosophers]” (Coates & Tognazzini, 2013; p. 13). At this point, we are reminded of a wonderful quote from C. Daniel Batson as he concluded a review of eight different ways scholars have used the term “empathy.” He says: “The best one can do is recognize the different phenomena, make clear the labeling scheme one is adopting, and use that scheme consistently” (Batson, 2009, p. 8). We hope we have been clear—and that we will continue to be clear throughout this article—about the different phenomena connected to blame and about our labeling scheme.

### The Target of Blame: Individual versus Group

Though much of the research in the blame literature has focused on blame of a particular individual, such as a spouse (Durtschi et al., 2011; Fincham & Bradbury, 1992), a child (Baumrind et al., 2010), an employee (Mitchell & Ambrose, 2007), a criminal offender (Gill & Pizzuto, 2022), or a generic other (Gill & Cerce, 2017; Monroe & Malle, 2019), blame has also been studied at the level of group perception (Alam et al., 2024; Bruneau et al., 2018,^2020^; Lickel et al., 2006). Such group-directed blame is exemplified in findings showing that, for example, individuals hate groups that commit violence against the ingroup (Halperin, 2008). They also hate groups that are perceived to have malicious intentions or to engage in recurring or extreme transgressions (Fischer, 2024). Relatedly, people are angry or contemptuous toward groups they perceive to act unjustly (Halperin, Russell, Dweck, et al., 2011; Tausch et al., 2011).

We conceptualize partisan animosity as an instance of *group-directed blame*. We deliberately avoid the term *collective blame* (e.g., Lickel et al., 2003), which refers to blaming an entire group for the actions of a small number of group members. Although such collective blame surely plays some role in the group-directed blame of partisan animosity (e.g., *Liberals are to blame for the murder of Charlie Kirk*), partisan animosity also involves blaming the outparty for beliefs or actions that one perceives as widespread within the outparty (e.g., *I think most conservatives dehumanize Mexican immigrants*). In short, then, we posit that partisan animosity—much, if not all, of it—can be understood as a form of group-directed blame. Although we conceptualize partisan animosity as group-directed blame, we assume that group-directed blame can color reactions to individual outparty members, just as group-level stereotypes and prejudice can color reactions to individual group members (Hamilton & Sherman, 1994; Ryan et al., 1996).

### The Target of Blame: Behaviors versus Mental Contents

#### Blame for Mental Contents is a Typical Form of Blame

Some might find it peculiar to think of partisan animosity as blame because it seems that blame is usually triggered by actions (see, e.g., all the work cited below in the section *The Psychology of Blame*) whereas partisan animosity seems to be triggered most often by perceptions of outparty *attitudes* or *beliefs* or *intentions*. This is less peculiar than it might seem at first. This is so for several reasons. First, many instances of partisan animosity *are* about actions—such as voting for the outparty in elections, circulating a petition in support of curtailing abortion rights, or making harsh or dishonest statements about the inparty on social media. Second, to take things to a deeper theoretical level, even when blame *appears* to be triggered by an action, it is *actually* triggered by the (assumed or inferred) underlying attitudes, beliefs, and intentions behind those actions. This is because blame is deeply rooted in social cognition and theory of mind (Malle et al., 2014), as we infer the mental states that drive actions and blame to varying degrees based on those mental state inferences. For instance, if a man interrupts a woman during a conversation, we might form a strong attitude of blame toward him because we assume *he believes that women are not worthy of respect*. However, if we later learn that he is deaf in one ear and did not realize she was speaking, our attitude of blame is tempered. Thus, the blame is not determined by his act (which is the same before and after we learn that he is deaf in one ear), but rather by the mental contents that seem to be reflected in that action.

Third, and perhaps most importantly, blaming others for their attitudes, beliefs, and intentions—even when those mental contents do not lead to action—is commonplace in social life. Although experimental social psychologists such as ourselves have tended to focus on blame for specific actions, moral philosophers (see Smith, 2013) have argued that we routinely blame others for their mental states in everyday life: *How could you want such a thing?! How could you believe such a thing?! How could you feel that way in this situation?! How could you feel nothing in this situation?!* On this view, the focus on attitudes, intentions, and beliefs in partisan blame is quite normal: *How could you want a world full of guns?! How could you want a world where the unborn are murdered?! How could you believe that poor people are just lazy?! How could you believe that opening the doors to millions of immigrants would be good for society?!.*

#### Why Does Blame for Mental Contents Play Such a Dominant Role in Partisan Blame?

Although blame for mental contents is a normal manifestation of blame, it is nevertheless arguably true that blame for mental contents is more central to partisan blame than to blame in other contexts. That is, partisan blame seems to be *overwhelmingly* directed toward the presumed mental contents of outparty members. Why would this be?

One reason has to do with the nature of political life today. That is, the outparty contains tens of millions of individuals spread across vast distances, and we observe the concrete actions of very few of them. Indeed, given the increasing geographic sorting of partisans into politically homogeneous communities (Brown & Enos, 2021), citizens today are less likely than ever to encounter outparty members and to directly observe their behavior. What we do observe, instead, is evidence of their attitudes, beliefs, and intentions through social media posts, polling data, online videos, and so on. Politics is, above all else, a game of words: arguments, tweets, podcasts, and opinion pieces that continuously convey information about what partisans think, believe, and feel. Naturally, because these mental contents are most visible to us, they become the primary target of our attitude of blame in the political arena.

A second reason is that blame often serves to protect what one perceives as a morally good community or society. When individuals believe that the outparty’s agenda would erode the moral fabric of their community, they blame in order to reduce the likelihood that this agenda will prevail. Before such feared outcomes materialize, blame tends to focus on the outparty’s “worldview”—its attitudes, beliefs, and intentions—because these mental contents are seen as the engines that will eventually produce concretely harmful actions and policies.

### Triggers of Blame: Political Parties Differ in Intuitions About What Counts as “Immoral”

How does our perspective relate to the highly influential idea of different moral foundations between liberals and conservatives (Graham et al., 2009, 2013)? Work on moral foundations suggests that liberals place more moral weight than conservatives on violations of the *Harm/Care* and *Fairness* domains of morality, whereas conservatives place more moral weight than liberals on violations of the *Loyalty*, *Authority*, and *Purity* domains of morality. The straightforward connection of this work to our own is its implication that, to a degree, liberals and conservatives will blame each other for different sorts of offenses. That is, liberals will blame conservatives for, say, lacking compassion for the vulnerable, whereas conservatives will blame liberals for, say, lacking sufficient love and respect for the nation and its traditions. In short, moral foundations theory implies partisan variation in the types of actions that will be perceived as wrong, and thus in the types of perceived immorality that will activate partisan blame. Other than this difference in triggering events, we expect that partisan blaming will otherwise look similar among liberals and conservatives. That is, they will rely on the same criteria for assessing blameworthiness (e.g., intentionality; Kollareth & Russell, 2022: Kupfer et al., 2020; Parkinson & Byrne, 2018) and they will experience the same sort of hostile attitude of blame toward those they perceive as wrongdoers.

### Connection to Existing Conceptualizations of Partisan Animosity

It is important to note that thinking of partisan animosity as blame is not a radical revision or repudiation of existing conceptualizations. Critically, however, our contribution is to make the link between partisan animosity and blame explicit and clear, and thus to highlight how the richness of the blame literature can be plumbed to generate novel solutions to the problem of partisan animosity. Indeed, although several existing conceptualizations align quite tightly with our definition of partisan blame, those conceptualizations do not use the term “blame” or draw insights from the blame literature. By explicitly making this link, our article integrates previously disconnected literatures and provides novel insights about how to ameliorate partisan animosity.

For example, our approach aligns with the conceptualization put forth by Finkel et al. (2020, 2024). Those authors argued that partisan animosity is driven by a combination of othering (viewing the outparty as fundamentally distinct from the inparty), aversion (strongly disliking and distrusting the outparty), and moralization (viewing the outparty as immoral). This sounds extremely similar to our concept of blame: Blame is aversion stemming from moralization, and it arguably involves “othering” (i.e., they are bad, we are good). Similarly, Hartman et al.’s (2022) definition of partisan animosity as “hostile thoughts, emotions, and behaviors directed at a political outgroup” is virtually identical to our definition of blame except for the fact that we emphasize the moralization of the hostility (i.e., *I hate them because they are morally despicable!*).

Furthermore, our conceptualization of partisan animosity as group-directed blame fits with established measures of partisan animosity (although, as with work in the preceding paragraph, the articles describing the measures do not explicitly refer to blame or draw from the blame literature). For example, the predominant measure of partisan animosity in existing literature is the “feeling thermometer,” which prompts Democrats and Republicans to rate their feelings toward the outparty on a scale from 0 (very cold) to 100 (very warm) (Iyengar et al., 2019). Arguably, this measure captures group-directed blame (albeit not well; see Finkel et al., 2024), as it assesses negative feelings toward the outparty, and negative feelings are integral to an attitude of blame. Notably, the feeling thermometer is not a strong predictor of anti-democratic attitudes (Voelkel et al., 2024). We would expect, however, that partisan animosity will show particularly strong connections to negative consequences (e.g., support for anti-democratic norms or violence) when the measure of partisan animosity more directly measures an attitude of intense blame rather than mere coldness (as with the feeling thermometer). Indeed, recent data from Finkel et al. (2024) are consistent with this expectation. Their measure of political sectarianism includes subscales for aversion, moralization, and othering. They found that the feeling thermometer and their own othering subscale (e.g., “I feel distant from [outparty members].”) were poor predictors of antidemocratic attitudes, whereas their aversion subscale (e.g., *I hate [outparty members]*) and moralization subscale (e.g., *[Outparty members] are immoral*.), which precisely capture an attitude of intense blame under our conceptualization, were strongly predictive of antidemocratic attitudes. Hating the outparty for their immorality—i.e., blaming them intensely—is a supremely destructive attitude.

### Summing Up and Moving Ahead

Though our blame-based framework is not a radical departure from existing conceptualizations of partisan animosity, its value lies in connecting the phenomenon of partisan animosity to a rich and generative unifying theoretical framework. That is, by thinking of partisan animosity as an instance of blame, we are invited to plumb the blame literature to understand how blame is aggravated and mitigated and to utilize these insights to better understand and alleviate partisan animosity. Whereas we feel certain that the understanding and amelioration of partisan animosity will be supported by conceptualizing partisan animosity as a manifestation of blame, we acknowledge that we cannot prove that every single instance of partisan animosity is blame. Indeed, we look forward to conversations about instances of partisan animosity that do not appear to involve blame and which clearly reflect a different form of intergroup negativity with its own distinct eliciting appraisals. Prior to plumbing the blame literature, we will first spend a moment illustrating how our conceptualization is highly compatible with prior work on the roots and accelerants of partisan animosity.

## Prior Work on the Roots and Accelerants of Partisan Animosity Merges Seamlessly With Our Conceptualization

Although our reconceptualization of partisan animosity as blame can stimulate novel insights about how to improve hostile political cultures around the globe, we also want to highlight that our approach is highly compatible with influential existing theories regarding the roots and accelerants of partisan animosity. These existing theories provide valuable insights into how partisan overblaming has come into being, why it has increased sharply over time, and how it is sustained within our current cultural and technological milieu. Below, we will review some of the major theories and research findings and describe how our approach merges seamlessly with each.

Perhaps the most influential account of the roots of partisan animosity is the social identity account (Iyengar et al., 2012, 2019; Mason, 2015, 2018; Van Bavel et al., 2024). Social identity theory (Tajfel & Turner, 1979) contends that the groups people belong to contribute to their sense of identity and self-esteem. Therefore, people desire to uphold positive views of their ingroups. This motivation for positive identity need not lead to blame of outgroups. In fact, one influential view is that social identity motives contribute to love for the ingroup more so than to hostility toward the outgroup (Brewer, 1999). Nevertheless, social identity motives surely can fuel an attitude of outgroup blame under certain circumstances, such as in an information environment in which examples of (supposed) outparty immorality are constantly in circulation (Brady et al., 2017; Rathje et al., 2021). Indeed, given that moral character assessment plays a dominant role in overall social evaluation (Goodwin et al., 2014), an attitude of blame toward the outgroup can serve as a powerful tool to establish a sense of ingroup excellence. Here, then, we integrate the social identity and blame perspectives with the notion that social identity concerns can motivate one to develop an attitude of blame toward the outgroup (Monroe & Malle, 2019; Munro et al., 2010; Reeder et al., 2005).

Importantly, political ideology has become increasingly central to people’s social identities in recent years, and cross-cutting identities that used to unite people across party lines (e.g., environmentalist; evangelical Christian) have merged with partisan identities (e.g., nowadays, nearly all evangelical Christians are Republicans; Huddy & Bankert, 2017; Mason, 2015, 2018; West & Iyengar, 2022). This contributes to both liberals and conservatives having sharper-than-ever cognitive representations of their political groups as mutually exclusive categories (Finkel et al., 2020; Iyengar et al., 2019). Such perceived mutual exclusivity of identity categories facilitates the development of animosity toward those in other categories (Bradley & Chauchard, 2022; Harteveld, 2021; Voelkel et al., 2024).

The preceding paragraphs highlight how social identity divisions can fuel group-directed partisan blame. The reader might take this to mean that social identity divisions are somehow “more fundamental” than blame for understanding polarization. We do not wish to make that claim. Rather, we would like to point out the plausibility of the notion that blame can fuel social identity divisions. To our knowledge, this possibility has not been explored in the literature. For example, a politically unaffiliated citizen might notice that, in the U.S. context, Republicans take a harsh stance against immigration and that President Trump’s ICE raids seem excessively aggressive and cruel. This citizen feels that this is morally wrong, and that one should have more compassion for immigrants. She develops an attitude of blame toward Republicans based on this perceived immorality. Consequently, she begins to identify as a Democrat because Democrats strike her as less blameworthy with regard to the immigration issue. In short, we believe—and we hope future research and theory will consider—that the causal arrows connecting identity and blame can flow in both directions.

Notably, social identity dynamics are intensified when the ingroup and outgroup are in competition (Campbell, 1965; Jackson & Esses, 2000; Sherif, 1966), and thus should be quite pronounced in the intrinsically competitive realm of partisan politics. All political parties want to rule. Of course, they can pursue this goal via positive means, such as developing policy proposals that appeal to a majority of citizens. But, they can also pursue this goal via negative means: Partisans can seek a competitive edge by convincing others that the outgroup is so despicable that they do not deserve to rule (e.g., Esses et al., 1998; Scheepers et al., 2002). That is, to the extent that Republicans can convince society that Democrats are morally despicable (or vice versa), they will have a competitive edge in elections. Thus, partisan overblaming is not merely in service of feeling good about the ingroup, it can also be strategically deployed to weaken the chances of the outgroup winning elections. This is another root cause of partisan animosity that fits our blame-centric conceptualization extremely well.

Another important theory of the roots of partisan animosity asserts that it begins with a real social cleavage (e.g., wealthy elites vs. struggling lower classes) that is exploited by political elites for personal gains such as political power (e.g., Banda & Cluverius, 2018; McCoy & Somer, 2019). The idea here is that, prior to elite manipulation, one social group is already blaming another, at least somewhat: *I think those rich folks don’t care much about average folks like us—so I don’t like them very much*. Elites exploit this pre-existing grievance by talking about it all the time and couching it in rhetoric that is carefully selected to intensify blame of the putative victimizer: *Your children are suffering every day because those wealthy folks refuse to pay their fair share of taxes! They don’t give a damn about you!* Thus, the elite rhetoric propels a “victim group” toward an ever more intense attitude of blame toward an “oppressor group.” After stoking such hyperblaming animosity, the elites then create a path to power for themselves by offering themselves as saviors for the victim group: *I will punish those bastards who mistreat you and I will get you the respect you deserve!*

To sum up, the roots of partisan overblaming lie in individuals and groups striving for positive identities and vying for political power for their group or for themselves. A hostile attitude of blame toward the outparty—*they are profoundly immoral and deserve nothing but contempt*—is a powerful tool for achieving these ends. Clearly, our blame-centric view coheres very nicely with these well-established theories.

Rather than focusing on roots of partisan animosity, other work focuses on accelerants that cause animosities to intensify and spread like wildfires. One crucial accelerant is misperceptions of the outparty (Ahler & Sood, 2018; Landry et al., 2023; Pasek et al., 2022; Yudkin et al., 2019). Increasingly, these are propagated via social media and via the 24/7 news media and thus can infiltrate more minds in less time than ever before in human history. Indeed, intensification of partisan animosity has been linked to increased availability of digital media (Lorenz-Spreen et al., 2023). Partisan media can further activate partisan identities and intensify hostile attitudes toward the outparty by framing the opposing side in moralized, condemnatory terms—often comparing them to Nazis or Communists (Berry & Sobieraj, 2013). Connecting this idea to our focus on blame, we note the profound moral overtones of many misperceptions. For example, whereas most Republicans (≈80%) agree that “Racism still exists in America,” Democrats believe that only half of Republicans agree with this (Yudkin et al., 2019). Similarly, whereas most Democrats (≈80%) agree that “Most police are good people,” Republicans believe that only a minority of Democrats agree with this (Yudkin et al., 2019). Of course, denial of racism is a serious moral offense in the eyes of Democrats and devaluing the police is a serious moral offense in the eyes of Republicans. So, clearly, these sorts of misperceptions will amplify an attitude of blame toward the outparty as they rapidly spread via digital media.

Relatedly, social media not only enable the rapid spread of blame-aggravating (mis)information, but they also appear to incentivize divisive content. For example, Brady et al. (2017) found that the presence of moral-emotional words in a tweet was a powerful predictor of retweeting and that the top three most virality-boosting words were *attack*, *bad*, and *blame* (see also Rajthe et al., 2021). Extending this work, Brady and Van Bavel (2025) have experimentally shown that partisans are sensitive to these incentives, as they prefer to share blame-filled content (e.g., *Can we please get Trump the hell out of office? His racist and misogynist ideology is disgusting; Democrats show no shame in exploiting political situations for votes. They need to stop pretending like they care about Americans*) over neutrally worded content expressing a similar point. Political elites also appear to be similarly motivated. Frimer et al. (2023) found that politicians have become increasingly uncivil on Twitter over time, and this is partially because their uncivil tweets are especially likely to be retweeted and liked. Obviously, such preferences for spreading blame-aggravating information will reinforce and inflame partisan animosity.

At this point, we have clarified the meaning of our claim that partisan animosity is a manifestation of blame and presented an analysis to show how our conceptualization merges seamlessly with prior work on the roots and accelerants of partisan animosity. Now, we will focus on the utility of our perspective. As a first step, we offer a general model of the psychology of blame.

## The Psychology of Blame

What are the triggering conditions of an attitude of blame? What sorts of cognitive appraisals determine the intensity of that attitude of blame? When an attitude of blame is intensely hostile, what factors can lead a person to downregulate hostility when expressing the attitude to others? These are the questions addressed by our model of the psychology of blame. See Figure 1 for a graphic depiction of our model.

**Figure 1.**
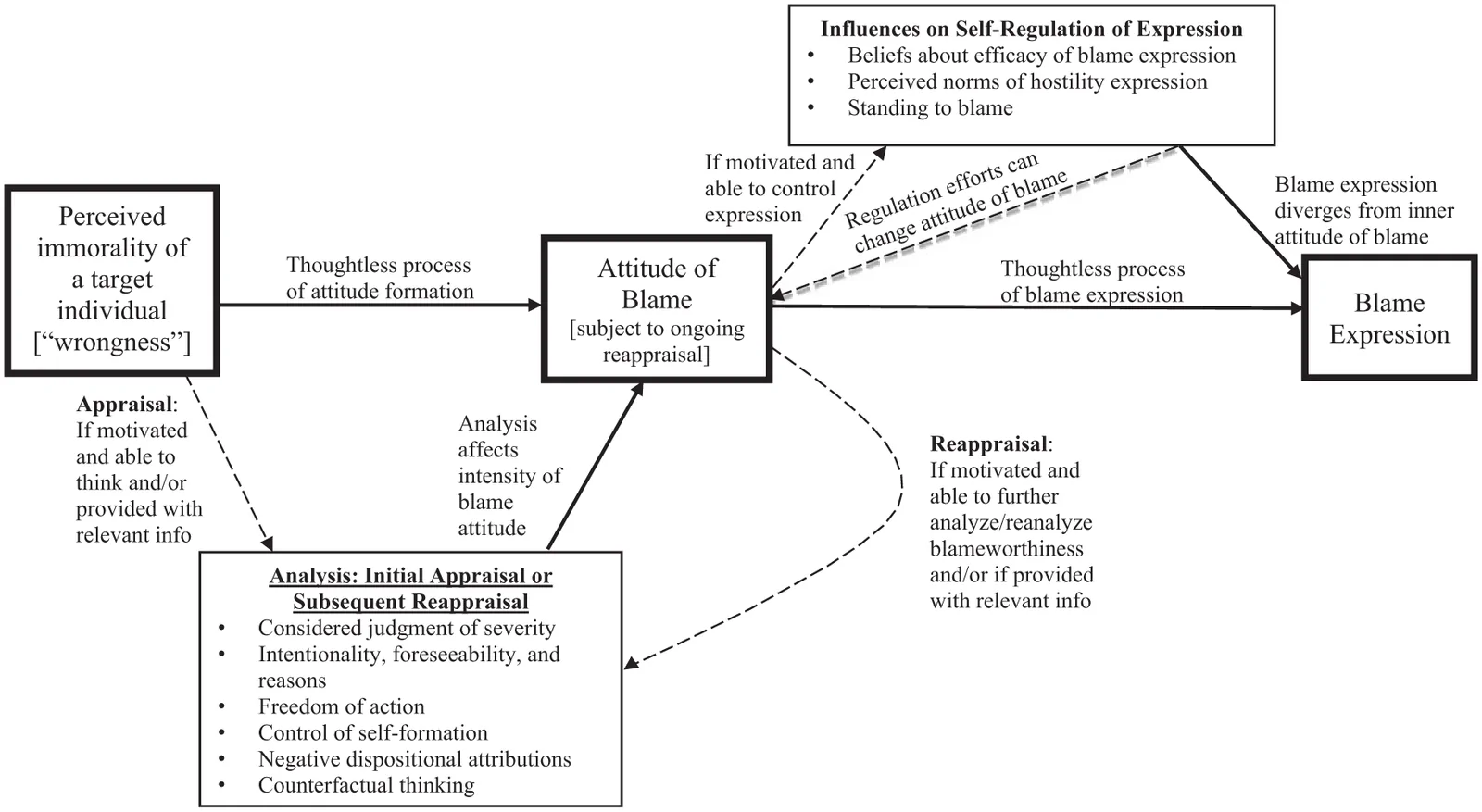
General model of blame. *Note*. The dotted lines indicate potential pathways that may or may not actually be followed by the perceiver.

Our key goal in the present article is to build a bridge between the literature on the psychology of blame and the phenomenon of partisan animosity. Thus, we will not spend a lot of time comparing our blame model to other blame models in the literature. We will point out, however, that our model of the psychology of blame synthesizes a highly diverse set of sources that have not been synthesized previously. Our model explicitly centers concepts from the most recent model of blame in the literature (i.e., *intentionality* and *reasons* from Malle et al., 2014), while also highlighting classic work from the tradition of attribution theory (Jones & Harris, 1967; Kelley, 1973; Weiner, 2006). Our model also integrates recent work focused on perceptions of free will (Gill & Cerce, 2017; Shariff et al., 2014; Vonasch et al., 2018) alongside work from cognitive psychology on counterfactual thinking (Kahneman & Miller, 1986; Spellman & Gilbert, 2014). Finally, like Malle et al. (2014), we distinguish between one’s inner attitude of blame and one’s outer expression of that attitude. However, whereas Malle et al. highlight how contemplating the public expression of blame—which is a serious matter—motivates us to be careful when forming our attitude of blame, our model highlights how various motivations can lead us to express blame calmly and respectfully even when we have an underlying intense attitude of blame. In describing these motivations for the down-regulation of blame expression, we draw on work from philosophy (Bell, 2013; King, 2024), organizational psychology (Geddes & Callister, 2007; Geddes & Lindebaum, 2020), and cross-cultural psychology (Matsumoto et al., 2008). In short, in building our model, we have thoroughly plumbed the literature on blame from various disciplines and subdisciplines to create a rich, integrative model that we believe can stimulate many ideas for ameliorating partisan blame.

As can be seen in Figure 1, the blame process begins with *perceived wrongness*: The perceiver detects that a target individual has done something morally wrong, or possesses attitudes, beliefs, or intentions that the perceiver considers morally wrong. The types of events that will be perceived as “wrong” are well-described by Haidt (2012; i.e., Five Foundations) and by Gray and his colleagues (Gray et al., 2012; Schein & Gray, 2018; perceived harm). Consistent with Malle (2021), we distinguish this initial perception of wrongness from the attitude of blame itself. This is a critical distinction, as it allows the perceiver to, say, continue judging something as wrong without despising or wanting to lash out at the individual or group that has produced the wrong. For example, in the context of partisan blame, this distinction allows for the following: *I still think that what the outparty is doing is wrong (and I will work hard to prevent it) but I do not hate them for it*.

Sometimes, perceived wrongness (*He lied to his wife*) will automatically elicit an attitude of blame (*I despise that jerk*) with no deliberation involved. This presumably happens because the perceiver makes implicit assumptions about the mental states and activities of the target (*He planned this lie for selfish reasons; This shows that he hates women)*, perhaps based on pre-existing impressions of the target’s dispositions (*He is often disrespectful to women*). However, perceivers do not always leap directly from perceived wrongness to an attitude of blame. Rather, sometimes they engage in reflection and analysis before forming such an attitude. This analysis may occur spontaneously (*I wonder why he told that lie? Is it possible that he had a good reason?*) or it may be triggered by the receipt of relevant information (*I heard that things weren’t going well for him at work. I bet he lied to his wife because he was trying to hide his struggles*). Whether the attitude of blame is formed automatically or analytically, blame *per se* reflects a hostile attitude toward the target person. This distinguishes it from perceived wrongness, which is an evaluation of an action, attitude, belief, or intention. As already noted above, attitudes of blame are graded: They can range from minimally hostile (*I’m slightly annoyed with him for lying to his wife, but I understand his reasons and I still believe he’s a decent guy*) to intensely hostile (*He disgusts me because of how he lied to his wife. I hope he fails in life!*).

We assume that, in the absence of thoughtful analysis, the intensity of blame tends to track perceived wrongness: mild wrongness brings mild blame, intense wrongness brings intense blame. Thoughtful analysis can, however, loosen this link. Much of the work on thoughtful analysis in the context of blame reveals how such analysis can temper the intensity of blame attitudes. This tempering effect is driven by changes in cognitive appraisals of several different determinants of blame intensity, which we will discuss in detail below. We also note that after an attitude of blame is formed—whether mindlessly or through thoughtful analysis—further thoughtful analysis (i.e., reappraisal) always remains possible. Such reappraisal, just like any initial appraisal that happens, can occur spontaneously or be prompted by the receipt of new information. Like an initial thoughtful analysis, reappraisal involves reflecting on the determinants of blame intensity described below. Thus, the model is iterative, allowing for reappraisal and adjustment of blame attitudes at any point in time (see Figure 1).

A large body of research has identified the specific factors that shape cognitive appraisals and reappraisals of blameworthiness and which thereby determine the intensity of blame attitudes. We now turn to these determinants of blame intensity in the following section.

### Determinants of Blame Intensity Potentially Considered During Appraisal or Reappraisal

#### Offense Severity

One powerful contributor to blame intensity is offense severity, which refers to the degree of perceived wrongness that is detected. For example, causing a victim to feel profoundly humiliated is more wrong than causing him to feel a twinge of embarrassment. Offense severity is tightly linked to the degree of harm experienced by victims. This is in line with the dyadic theory of morality, which posits that actions are viewed as wrong in proportion to their perceived harm (Gray et al., 2012; Schein & Gray, 2018). Indeed, a number of studies have found that the more harm a victim experiences, the more blameworthy the perpetrator is perceived to be (Boon & Sulky, 1997; Gray et al., 2012; Gray & Wegner, 2011; Lerner & Miller, 1978; Miller & Vidmar, 1981; Schein et al., 2016; Schein & Gray, 2018; Vidmar & Crinklaw, 1974; Wortman, 1976). Similar to Malle et al. (2014), we assume that perceptions of wrongness are generally rapid, automatic, or “intuitive” (Haidt, 2012). Our model also emphasizes, however, that thoughtful analysis or reappraisal of severity is possible (*Was it really that bad? Did it cause as much harm as I initially thought?*) and that this can change one’s attitude of blame.

The importance of offense severity can also be seen at the group-level that interests us. For example, Longo et al. (2014) found that Palestinians who perceived Israeli checkpoints in the West Bank as *humiliating* (severe offense) showed a stronger attitude of blame toward Israel than did Palestinians who perceived the checkpoints as *creating uncertainty about travel times* (moderate offense).

#### Intentionality and Reasons

Perceived intentionality and perceived reasons, which are integrally related, are also major contributors to blame intensity (Malle et al., 2014). *Perceived intentionality* refers to whether the actor engaged in the action *knowing* what the consequences would be and *desiring* those consequences (Malle & Knobe, 1997). Across cultures, intentional violations elicit more blame than unintentional violations (McNamara et al., 2019), even in scenarios where unintentional actions yield severe negative consequences (Cushman, 2008). This propensity for stronger blame responses toward intentional violations holds true across various moral domains (e.g., harm and purity; Kupfer et al., 2020). A closely related concept to intentionality is *perceived foreseeability* (Fincham & Jaspars, 1980), because people cannot have intended an outcome that they did not foresee. Yet, importantly, they can be blamed for unforeseen harm if a perceiver believes that they *could have* or *should have* foreseen it (Lagnado & Channon, 2008; Malle et al., 2014).

Once intentionality has been established, individuals may move forward with evaluating the reasons that motivated the transgressor’s intentional bad action (Malle et al., 2014). *Perceived reasons* are what a perceiver infers to be the *particular intentions* behind an action. Considering a perpetrator’s reasons is integrally tied to assessments of blameworthiness because reasons determine the meaning of actions (Binder, 2000; Scanlon, 2008), thereby revealing aspects of the violator’s character, such as their motives, beliefs, and attitudes (Malle, 2004; Stueber, 2009). Whether or not a given reason aggravates or mitigates blame depends on social norms (Alexander, 2009; Shaver, 1985), and characteristics pertaining to both the blamer and the blamed (Polman, Pettit, & Wiesenfeld, 2013; Riordan et al., 1983; Tetlock et al., 2007). Generally speaking, blame-aggravating reasons establish that the action was committed based on immoral motives (e.g., selfishness, callousness, racism), whereas blame-mitigating reasons establish that the action was committed based on moral motives (e.g., protecting others, creating fairness). As it so happens, even children demonstrate the capacity for considering reasons. For example, Darley et al. (1978) found that children as young as 5 years old were inclined to recommend less punishment for a child who had harmed another child if the act was perceived to have proceeded from an intention to protect others.

Although we generally think of intentions and reasons as existing in the minds of individuals, these concepts are sometimes relevant to group perception, too. This is particularly likely when a group is perceived as entitative (Newheiser et al., 2012; Sacchi et al., 2009) or when people simply assume that reasons of a few group members reflect the reasons of all or most group members (Allison & Messick, 1985)

#### Freedom of Action

Another major contributor to blame intensity is perceptions of the transgressor’s freedom of action, called controllability in Weiner’s classic work (Weiner, 2006). We change the name based on Gill and Cerce’s (2017) finding that lay perceivers use multiple concepts of controllability when assigning blame. Here, violators evoke a stronger attitude of blame to the extent they are perceived as doing wrong while having an unconstrained (free) ability to do right. In a classic study, Weiner and Kukla (1970) demonstrated the impact of perceived freedom of action in evaluations of student performance. They discovered that teachers were more critical of students who did not expend effort (although they could have) than of students with equally low exam scores but who had low IQs (who cannot choose to be smarter).

Freedom of action has been manipulated in a variety of ways, such as through narratives involving biological impairment and determinist arguments about free will. For example, Gill and Cerce (2017) exposed participants to (fictitious) biological information about double-murderer Robert Harris. The information explained that Harris had suffered brain damage from a traumatic birth. Relative to a control group, participants exposed to this information showed tempered blame reactions to Harris, and this effect was driven by the perception that Harris had limited freedom to act otherwise. Similarly, Shariff et al. (2014, Studies 2–3) presented people with essays or neuroscientific articles arguing against free will. Then, they had participants read a story about a violent offender and were asked to judge what his prison sentence should be after he underwent a nearly 100% effective rehabilitation program (this latter detail was introduced to isolate participants’ desire for retribution from their desire for offender transformation/deterrence). They found that anti-free will arguments reduced support for retributive punishment against the offender.

Whereas work in the preceding paragraph reduced perceived freedom of action by reducing the sense that the actor is making any choice at all (i.e., via determinism information), other work focuses on how perceived freedom of action can be diminished when the actor clearly *is* making a choice but some of the options come with such dire side effects that no reasonable person would select those options. In the extreme case, an actor has diminished freedom of action when he “chooses” to say something because there is a gun to his head. As a more realistic, everyday example, Vonasch et al. (2018) found that a CEO is rated as having greater freedom of action than is a subordinate worker. This is because the subordinate’s role in the organization requires that he “do what he is told” (which might not be identical to what he wants to do), whereas the CEO’s role empowers her to “do what she wants.” Indeed, a subordinate could face termination for failing to do what he is told, whereas such external constraints on action are less of a concern for the CEO. This means that the subordinate has less freedom of action than the CEO. Thus, here, perceived freedom of action depends on perceptions of whether the actor is “forced” to make certain choices because alternative choices would have dire side effects (e.g., unemployment).

People also perceive the freedom of action of groups, which, in turn, impacts their group-directed attitude of blame. For example, Lickel and colleagues (2003) found that the extent to which people perceived the parents and peer groups of the Columbine High School shooters as either freely encouraging or failing to prevent the shootings, the more they were blamed for the massacre. Furthermore, those who view criminal offenders in general as having ample freedom of action are significantly more likely to support punitive criminal justice policies (Gill et al., 2021; Study 5).

#### Control of Self-Formation

Control of self-formation, the perceived extent to which immoral actors are believed to have created their own immoral character, is another major contributor to blame intensity. Gill and Cerce (2017) have shown that diminished perceptions of control of self-formation decrease blame, and that this happens independent of perceptions of freedom of action. Thus, control of self-formation and freedom of action are two distinct notions of agent control. In their experiments, Gill and Cerce reduced control of self-formation perceptions through historicist narratives, story-like accounts of a person’s character development centered on the impact of formative life experiences. In an experiment involving an office bully named James, participants were presented with historicist narratives (or not) about James. One narrative described how James had been bullied by his father during his upbringing, an experience that contributed to James becoming a bully himself. In another experiment, the narrative detailed James’s over-indulgent parents, who played a role in his developing arrogance and a lack of respect for others. Across both narratives (i.e., abuse narrative, spoiled narrative), the historicist narratives reduced blame of James for his bullying behavior, and this effect was driven by diminished perceptions of control of self-formation. Interestingly, the historicist narratives did not reduce perceived freedom of action: Participants in the narrative and no narrative conditions strongly and equally believed that James could choose to refrain from bullying.

Gill et al. (2021) showed how historicist narratives and perceived control of self-formation also function at the group-level. Participants completed a measure of historicist beliefs regarding criminals as a collective, that is, the extent to which they believed criminals, in general, develop criminogenic traits based on unfortunate formative experiences. A test of mediation indicated that those who endorsed historicist beliefs regarding criminals as a collective also reported reduced perceptions of criminals’ control of self-formation, which, in turn, was associated with less support for harsh criminal justice policies.

#### Negative Dispositional Attributions

Another critical contributor to blame intensity is negative dispositional attributions. Dispositional attributions involve an inference that an actor’s perceived immorality is reflective of a broader bad character: She mistreats her co-worker *because she is a callous person* (as opposed to, say, *because her co-worker betrayed her in a vicious way*). This idea harkens back to the classic person versus situation dichotomy of attribution theory (Heider, 1958; Jones & Harris, 1967; Kelley, 1973; see Moskowitz & Gill, 2013, for a review).

One illustration of the role of dispositional attributions in blame comes from Witte et al. (2006), who examined blame of a husband who physically assaulted his wife. The researchers varied whether the victim had been verbally aggressive prior to the assault, and whether the perpetrator had a history of violence. Results revealed that perpetrator blame was heightened when the victim had not engaged in verbal aggression prior to the assault. Presumably, this stemmed from a dispositional attribution: A husband who attacks his peaceful wife is a callous monster. Additional results suggested that victim blaming was heightened when the victim had been verbally aggressive prior to the assault and the perpetrator had never harmed anyone before. Again, this seems to reflect the operation of a dispositional attribution: A wife whose verbal aggression elicits aggressive behavior from a never-before-aggressive husband must be very cruel.

Halperin’s (2008) research on the Israeli–Palestinian conflict has demonstrated the consequences of dispositional attributions at the group level. He found that the extent to which individuals perceived acts of terrorism as reflecting an evil outgroup character predicted hatred of the outgroup. These feelings of hatred, in turn, predicted support for political and social exclusion, and even violence, against the outgroup.

#### Counterfactual Thinking

When a negative outcome occurs, observers are likely to engage in counterfactual thinking: *How might this negative outcome have been avoided?* (Kahneman & Miller, 1986; Roese, 1997). In the context of blame, counterfactual thinking involves mental simulation of alternative worlds that undo a harm that was done in the real world. The counterfactual thinker attends to which aspects of the world, when varied, seem most likely to prevent the harm. Research suggests that such counterfactual thinking tends to focus on undoing controllable actions by human agents (*If only he had not done X*) or on undoing abnormal conditions (*If only she had done it the way she normally does*; Alicke et al., 2015). An attitude of blame toward an actor is intensified to the extent that the counterfactual suggests that the actor could have easily done something differently and that this would have greatly reduced the probability of the harm (e.g., *If only he had checked the door before going to bed. . .*; Alicke et al., 2015).

Spellman and Gilbert (2014) discuss findings from Nadler and McDonnell (2011) through this counterfactual lens. Nadler and McDonnell reported a study in which participants learned about an explosion in a man’s shed that killed one of his neighbors. An important contributor to the explosion was the oxygen tanks stored in his shed. Results revealed that the man was blamed more for the explosion when he stored the oxygen tanks for business purposes than when he stored them because his daughter required oxygen for a chronic respiratory disease. Spellman and Gilbert argue that this pattern happened because it is easy to imagine a world in which the businessman stores his oxygen tanks elsewhere (e.g., in a warehouse connected to his business), whereas it is difficult to imagine a world in which the father who is caring for his daughter stores her desperately needed oxygen tanks elsewhere. Another study linking counterfactual thinking to blame was conducted by Branscombe et al. (2003). Those authors found that victims of sexual assault suffered more self-blame to the extent that they generated counterfactual worlds in which their own choices could have prevented their victimization (e.g., *I should’ve stayed home that night*).

#### Informational, Cognitive, and Motivational Biases

Although some prominent theorists assert that blamers succumb to a variety of blame-aggravating cognitive and motivational biases when assessing blameworthiness (Alicke, 2000, 2008; Clark et al., 2014), others argue that blamers are generally fair and rational agents who process blame-relevant information in a balanced, even-handed manner (Malle et al., 2014; Monroe & Malle, 2019; Monroe & Ysidron, 2021). Indeed, these latter theorists argue that there are alternative explanations for studies supposedly demonstrating bias and that the alternative explanations point to rational information processing under impoverished information conditions (Malle et al., 2014).

One body of research that purports to demonstrate bias is work on how blame-related inferences are (supposedly) distorted by spontaneously activated negative affective reactions to perceived harm or to those who produce such harm. As an example, Alicke (1992) conducted an experiment in which participants read one of two scenarios involving a man who sped home during a rainstorm, resulting in an accident that harmed others. In one scenario, participants were informed that the man was speeding home to hide cocaine from his parents (a character who should spontaneously activate negative affect), whereas in the other, they were told he was rushing home to hide an anniversary gift for his parents (a character who should not spontaneously activate negative affect). Even though participants received identical information regarding the accident in both conditions (e.g., he was speeding, there was a rainstorm), participants viewed the cocaine user as more responsible for the accident than the anniversary gift giver. Alicke argued that this reflects a bias in which exaggerated blame is pinned to those who activate negative affect (the drug dealer). Malle et al. argue that it’s unclear whether this work demonstrates such an affect-driven bias. Rather, they suggest that participants might reasonably assume that a person who accidentally leaves cocaine on his parent’s table drives more recklessly than a person who remembers to get his parents an anniversary gift. If so, then it is rational and defensible to view the cocaine user as more responsible for the accident.

Another type of (supposed) bias centers on how blame-related cognitive appraisals are affected by pre-existing negative perceptions of the transgressor’s character [Note: In the preceding paragraph, Malle et al. (2014) interpreted Alicke’s (1992) data as reflecting this phenomenon rather than the affect-driven bias posited by Alicke.] Here, rather than blameworthy acts leading to perceptions of bad character, pre-existing perceptions of bad character lead to perceiving an actor as more blameworthy (Nadler & McDonnell, 2011; Pizarro & Tannenbaum, 2012). That is, pre-existing dispositional attributions (*I know from past experience that she is selfish*) can bias perceptions of the actor’s intentionality, reasons, and so on, for an act that is perceived later (*This morning, I’m sure she intentionally left the clogged toilet for someone else to deal with*). As an example, Nadler and McDonnell (2011) had participants read about a case where a woman's dogs killed a child after escaping from their owner’s fenced yard. The dog owner was viewed as more responsible for the child’s death when she was described as having an unlikable, disreputable personality than when she was described as having a charming, prosocial personality. Malle et al. (2014) argue that it is not clear that this should be called a “bias.” Rather, they suggest, as in the preceding paragraph, that participants might simply be making the defensible assumption that a “bad person” probably exerted less effort to ensure that her dogs were contained than did a “good person” (see Nadler, 2012 for a rebuttal). Whether or not one wishes to use the label “bias,” we think this phenomenon likely plays an important role in partisan contexts: If one already believes that the outparty is full of “bad people,” then the intensity of one’s blame attitude toward them for new offenses will be stronger than if one had a more favorable pre-existing impression of them. This will happen because their “wrongness” will be more likely to be perceived as intentional, done for bad reasons, and so on (i.e., cognitive appraisals of their blameworthiness are influenced by the negative character impression).

Gill and Ungson (2018) provided evidence for motivated bias in blame. In one study, they had participants read about double-murderer Robert Harris. To manipulate motivation, some participants were instructed to act as prosecuting attorneys and others as defense attorneys. As an additional manipulation, some participants learned only about Harris’s murders whereas others also read a historicist narrative about Harris’s brutal childhood. After receiving information about Harris, the “attorneys” were asked to present arguments that supported either harshness (for prosecutors) or mercy (for defenders). After writing out their arguments, participants were told that the role play was over. Next, they were given some filler questionnaires and then they were asked for their *personal opinions* about Robert Harris. Results indicated that, in the condition where Harris’ brutal childhood was described, those who had previously been defenders blamed Harris less than did those who had previously been prosecutors. Coding of the arguments participants made while enacting their role as either defender or prosecutor suggested that motivated self-persuasion had occurred: Former defenders blamed Harris less than former prosecutors to the extent that, during the time they were motivated to make arguments for leniency, they framed Harris’ brutal history as severely limiting his freedom of action.

### Determinants of Blame Expression

Blame expression happens when a blamer communicates her attitude of blame to the transgressor or to others (e.g., criticizing a selfish friend to his face and/or in front of his family). In cases where a blamer simply expresses her true attitude, all the factors that impact blame intensity (discussed above) will also impact the intensity of blame expression (see direct path from *Attitude of Blame* to *Blame Expression* in Figure 1). But, of course, people do not always express their true attitudes. Sometimes, one has a strong inner attitude of blame (*I’m so angry at her!*) but, on the outside, it is not apparent. The reason is that blamers can down-regulate their blame expression, exerting effort to, say, speak calmly even while experiencing inner outrage. Of course, it is also true that blame expression can be up-regulated in the service of particular goals (e.g., performative outrage to signal partisan loyalty). Because our emphasis in this article is on reducing hostility, the factors below are relevant to motivation to down-regulate rather than up-regulate blame expression (see the path from *Attitude of Blame* to *Influences on Self-Regulation of Expression* to *Blame Expression* in Figure 1). Note that the model also assumes it is possible for the motivation to down-regulate blame expression to temper the underlying attitude of blame (see dotted line from *Influences on Self-Regulation of Expression* to *Attitude of Blame* in Figure 1). Indeed, lowering our inner feelings of hostility toward a person—rather than merely trying to hide them—is surely an effective strategy for communicating more constructively with them.

#### Beliefs About the Efficacy of Blame Expression

People are sometimes motivated to down-regulate blame expression based on beliefs they have about the *perceived efficacy of blame expression*. That is, some people believe that outward expressions of angry condemnation will produce no benefit or might even make a situation worse (*If I yell at my wife, she will lose respect for me and become more oppositional*). Beliefs about the efficacy of blame expression—*Is full-throated expression of blame likely to facilitate my goals (e.g., to persuade this person to be kinder)?*—are related to what emotion regulation theorists label instrumental motives for emotion regulation (Geddes & Lindebaum, 2020).

Gill et al. (2025; Study 2) provided evidence relevant to this idea. They examined the impact of a blame efficacy workshop on blame expression among first-year college roommates. Roommate pairs were randomly assigned to an in-person workshop that described the inefficacy of excessive blame expression or to a control (no workshop) condition. In the workshop, roommate pairs learned about, for example, Durtschi et al.s’ (2011) longitudinal research showing that angry blame expression during couple conversations was associated with diminished marital quality over time. Participants also learned tools to communicate more effectively when their roommates behaved badly. A follow-up survey later in the semester showed that the workshops successfully reduced belief in the efficacy of harsh blame (e.g., “Harshly blaming my roommate is likely to improve his/her behavior”) and these reductions in blame efficacy beliefs were associated with reductions in expressed hostility toward one’s roommate (Alam, 2023).

#### Beliefs About the Normative Appropriateness of Blame Expression

People will also down-regulate blame expression based on *perceived norms regarding appropriateness of anger expression*. That is, some people believe that outward expressions of angry blame are immoral. Here, the belief is not that blame expression is counterproductive, but rather that it is *morally wrong*. Because people want to be moral (Prentice et al., 2018), the belief that blame expression is counter-normative will motivate down-regulation of blame expression. Beliefs about the appropriateness of anger expression are not merely an individual matter, as there are often broad cultural beliefs about such matters in organizations (Geddes & Callister, 2007) and in nations (Matsumoto et al., 2008). Beliefs about the appropriateness of anger expression—*Is full-throated expression of blame consistent with my notion of what it means to be a good person?—*are related to what emotion regulation theorists label eudaimonic motives for emotion regulation (Geddes & Lindebaum, 2020)

#### Standing to Blame

Standing to blame is a concept that has received abundant attention from philosophers (Bell, 2013; King, 2024), but only limited attention from social psychologists (Ghezae et al., 2025). One’s standing to blame is challenged whenever one is asked: *Who are you to judge?* When people recognize that they lack standing to blame, they are more likely to keep their hostile criticisms to themselves (i.e., down-regulate their blame expression).

Whether one has standing to blame has been argued to depend on a variety of factors (King, 2024). These include whether one has engaged in similar wrongdoing in the past (a dishonest politician lacks standing to blame another politician for dishonesty, although politicians seem uniquely unlikely to recognize this!), whether one has a stake in the other’s behavior (e.g., other people’s private lives are none of our business), and whether one is part of the same moral community as the target of one’s criticism (e.g., modern humans lack standing to blame humans who inhabited very different moral communities centuries ago). Ghezae et al. (2025) examined folk intuitions regarding the standing to blame and their results supported many of King’s theoretical suggestions. Presumably, recognition that one lacks standing to blame can lead one to refrain from expressing blame.

Interestingly, Bruneau and et al.’s (2018, 2020) collective blame hypocrisy intervention may have prompted participants to reflect on their standing to blame. In their 2018 study, White Americans were asked to what extent they blamed themselves and White people as a group for acts of White supremacist terrorism committed by individuals such as Dylan Roof, Anders Breivik, and Wade Page. Naturally, White Americans did not view themselves as responsible for the acts of these extremists. This exercise was found to decrease the level of blame White Americans assigned to individual Muslims and to Muslims as a whole for Islamist terrorist attacks. By having participants reflect on their potentially hypocritical stance in this context, they arguably prompted participants to reflect on their standing to blame (e.g., *Who am I to blame all Muslims when people from my group have also engaged in horrific violence?*).

## Group-Directed Partisan Blame: Toward Understanding and Transforming an Affectively Polarized Political Sphere

In the remainder of this article, we will focus on how the blame model just described can help us understand and, ideally, transform our hyper-hostile political sphere into an arena where disagreements or different political priorities can exist without so much gratuitous hostility surrounding them. First, we will show how the blame model provides a unifying framework for interpreting existing animosity-reduction interventions. Then, we will show how the blame model is generative and useful for brainstorming potential future interventions.

Before proceeding, we wish to highlight some important ways in which the processes shaping group-directed partisan blame (Figure 2) are likely to differ from those shaping individual-directed blame (Figure 1). Accordingly, Figure 2 re-packages the blame model from Figure 1 with a few important revisions designed to make the model especially relevant to the context of partisan blaming.

**Figure 2.**
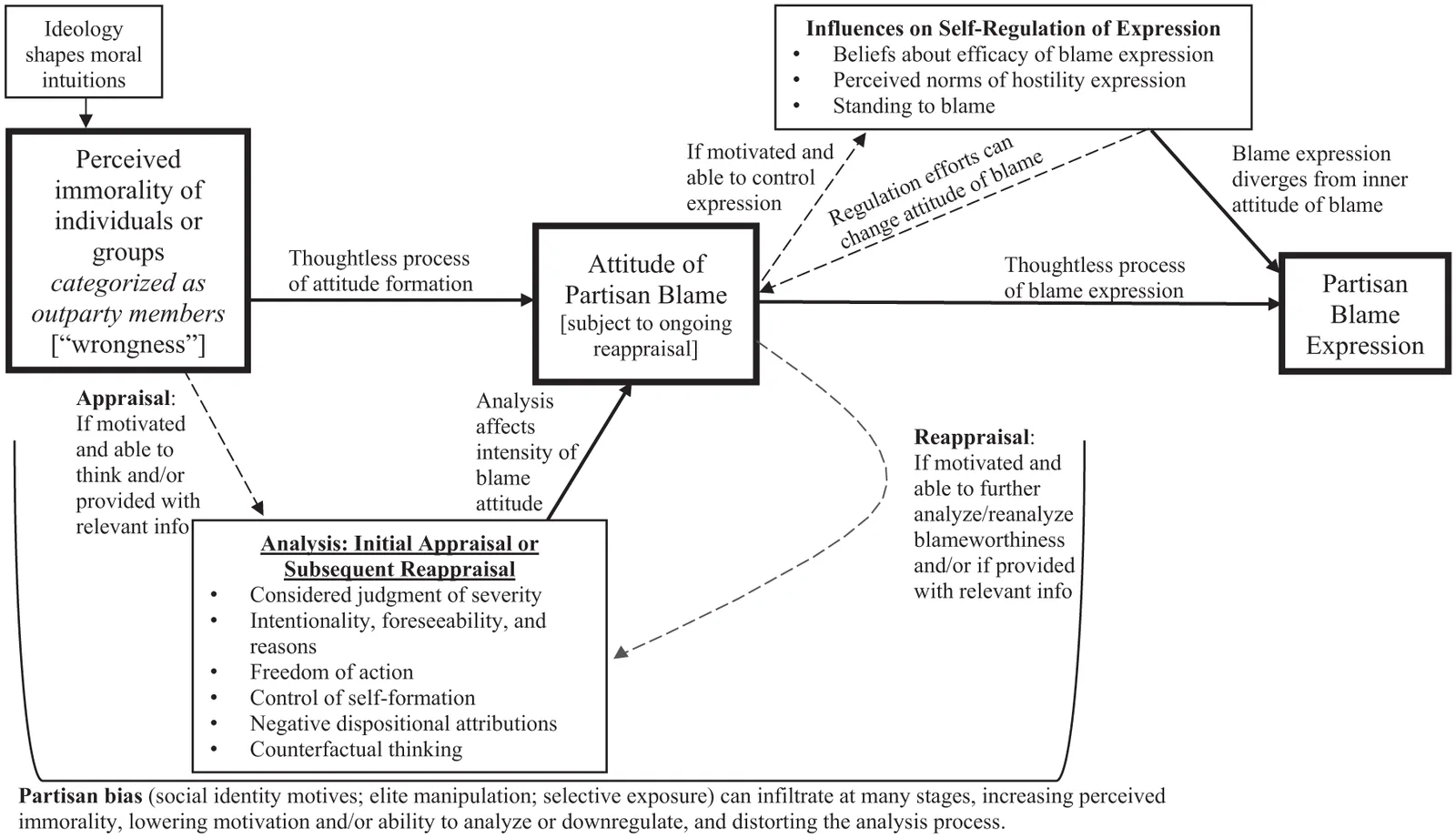
Model of blame in the Partisan context. *Note*. The dotted lines indicate potential pathways that may or may not actually be followed by the perceiver.

Several things are worth noting as we move from the individual-directed blame model of Figure 1 to the group-directed partisan blame model of Figure 2. First, although we had a section on *Informational, Cognitive, and Motivational Biases* when discussing Figure 1, we also cited prominent scholars who argue that there is not much evidence supporting the prevalence of such biases at the individual-level. In contrast, it seems certain that bias will be a powerful force shaping group-directed partisan blame (Ditto et al., 2019). Indeed, we have already discussed how social identity needs and competitive goals—both central to partisan contexts—create a motivation to impugn the outparty. To elaborate, this motivation to impugn should, theoretically, be influential at multiple stages in the partisan blame process (see bracketed area at the bottom of Figure 2) by: (a) Contributing to exaggerated perceptions of perceived immorality [e.g., via selective exposure to “news” about the outparty (Berry & Sobieraj, 2013) or cognitive distortions in one’s perceptions of such news (Kahan et al., 2012)], (b) creating a tendency to leap immediately from perceived wrongness to intense partisan blame, bypassing any thoughtful, fair-and-balanced analysis of blameworthiness (e.g., because one is motivated to impugn rather than to be fair to the outparty; Monroe & Malle, 2019), and (c) distorting whatever analysis of blameworthiness one does engage in (e.g., assuming that the reasons behind outparty actions are despicable even when equally plausible, less despicable reasons are possible; Waytz et al., 2014). Because of these phenomena, Figure 2, unlike Figure 1, explicitly includes bias as a factor in the model, a factor that will be influential at all stages that fall within the “bias bracket” at the bottom of the figure. We suspect that the fact that partisan blame is often about mental contents rather than concrete actions—a fact discussed above—might facilitate the operation of bias in partisan blame. The reason is that beliefs are less observable and more ambiguous than actions. While actions can be directly witnessed, beliefs are internal and must be inferred from limited cues, such as speech, writings, and group affiliations. This ambiguity may make beliefs especially vulnerable to partisan misperceptions and exaggerations that heighten blame.

Another important difference as we shift to a focus on group-directed partisan blame is that the model in Figure 2 begins with *perceived immorality of individuals or groups categorized as outparty members* (whereas Figure 1 begins with *perceived immorality of a target individual*). Thus, Figure 2 applies to any case where outparty members are processed qua outparty members. We suspect that, in a partisan environment, outparty members (especially when doing wrong things) are typically processed qua outparty members. That is, Republicans are likely to respond to an obnoxious Democrat by thinking, *Typical Democrat!*, rather than, *He’s only an individual—don’t generalize!* Of course, Democrats will react to an obnoxious Republican in the same way. Furthermore, whereas Figure 1 illustrates a process that will be triggered by the perception of a single target individual, Figure 2 illustrates a process that will be triggered a much more diverse array of inputs: Outparty “wrongness” can be perceived in speeches given by liberal or conservative politicians, societal impacts of policies enacted by liberal or conservative politicians, arguments made by liberal or conversative pundits, behaviors of liberal or conservative attendees at a protest (or riot, depending on your partisan perspective), social media posts by liberal or conservative family members, social media posts by liberal or conservative strangers, news articles about the public or private behavior of liberal or conservative individuals ranging from elites to ordinary citizens, and so on. That is, an attitude of partisan blame can be intensified—or tempered—by information about outparty members broadly construed.

Now, having made these points about unique features of group-directed partisan blame, we turn our attention to the literature on interventions to reduce partisan animosity. Our focus will be on how a diverse array of interventions can all be interpreted in terms of concepts from our model of the psychology of blame.

## Reconceptualizing Existing Partisan Animosity Interventions: How Effective Interventions Target the Determinants of Blame Intensity or Blame Expression

In the preceding sections, we offered a rich set of concepts relevant for understanding when blame attitudes or blame expression will be intensely hostile and when they will be tempered. Now, we will demonstrate how the burgeoning research literature on interventions to reduce partisan animosity can be understood in light of those concepts. That is, upon inspection of the diverse interventions in the literature, we believe that their animosity-reducing effects can be readily interpreted within our psychology of blame framework. That is, the interventions reduce partisan overblaming by targeting one or more of the determinants of blame or blame expression described above. Figure 3 presents a condensed version of the model from Figure 2, retaining only those features that are relevant for understanding or generating interventions to reduce partisan overblaming. Whereas the model in Figure 2 speaks to both the origins of partisan overblaming how it might change over time, Figure 3 takes the existence of intense attitudes of partisan blame as a given and focuses solely on how those attitudes or their expression might be tempered via third-party interventions. Figure 3 is the most relevant figure for understanding the remainder of this article. All the interventions below—whether actual and potential—are based on concepts that can be found in the two boxes in Figure 3 that are located between *Intense Attitude of Partisan Blame* and *Tempered Attitude* or *Tempered Expression*.

**Figure 3.**
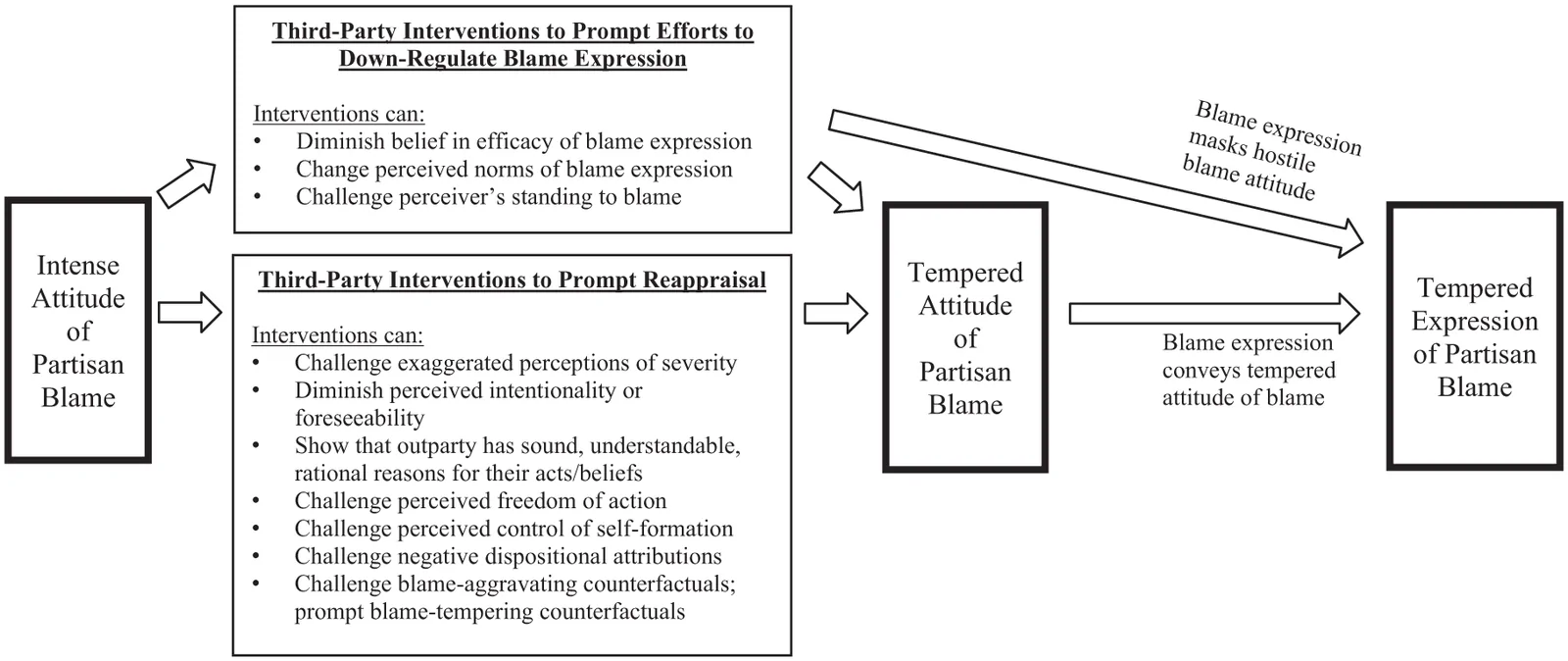
Condensed model of partisan blame for understanding and brainstorming third-party interventions.

We must add an important caveat prior to this exercise: The various intervention studies were not designed with blame concepts in mind and thus do not always measure the precise set of concepts we would like. Therefore, confirmation of our interpretations below awaits further research. Also, although we will often focus on one particular blame factor that strikes us as most relevant for understanding the efficacy of a given intervention, it is surely the case that most of the interventions—because they involve presentation of a rich array of information—target more than just one blame factor. We hope that our review here will stimulate future research that more carefully tests a variety of blame-relevant mechanisms to illuminate precisely why various interventions are effective.

### Interventions Focused on Determinants of Blame Intensity

#### Interventions that Target Offense Severity

From our review of the literature, we find no existing interventions that clearly target perceptions of offense severity. We find it surprising that this factor has not been studied in the context of partisan animosity, given that everyday political rhetoric seems highly focused on exaggerating perceptions of the amount of harm caused by outparty members (e.g., the outparty is *destroying our nation*, *eviscerating our constitution*, and *allowing us to be overrun by immigrant invaders*). We strongly encourage future research to test interventions to reduce partisan animosity via reducing perceptions of offense severity. In the proposed interventions section of this article (below), we will offer ideas for such interventions.

#### Interventions that Target Intentionality and Reasons

Some interventions lower animosity by changing perceptions of the intentions or reasons behind outparty policy stances. For example, Stanley et al. (2020) assigned participants to read about one of three issues (requiring standardized testing in primary schools, mandatory use of police-body cameras, animal testing for scientific purposes) and asked them whether they supported or opposed the issue stance. Then, they randomly assigned participants to one of three conditions: opposing reasons, supporting reasons, or control. In the *opposing reasons condition*, participants were provided with compelling reasons to believe what their opponents believe. For example, if a participant was against animal testing, they would learn that animal testing has been crucial in discovering many life-saving treatments for debilitating diseases. In the *supporting reasons condition*, participants were provided with reasons that supported their own stance (e.g., *Alternative methods eliminate the need for animal testing*). In the *control condition*, participants were not given any reasons. Results revealed that participants in the opposing reasons condition developed more positive views of the morality and intelligence of their opponents. Seeing that opponents adopt beliefs based on compelling reasons reduces blame for their possession of such “wrong” beliefs.

Similarly, Kubin et al. (2021) had liberals and conservatives read about how political opponents adopted their political beliefs based either on personal experiences of harm or on factual information. In one experiment, they had participants read about individuals who held opposing views to them on policies related to either coal, guns, or tax regulations. Some participants learned that the opponent held her opinion because of personal experiences of harm (e.g., coal regulations led her to lose her job) or because of factual information (i.e., information she learned while reading about coal regulations). The results revealed that when partisans were exposed to opponents who adopted political stances based on personal experiences of harm, they judged those opponents as more worthy of respect and more rational than opponents who relied only on facts. In short, partisans blame their opponents less when they come to see them as rational agents who are trying to avoid personal harm.

As another example, Waytz et al. (2014) randomly assigned Democrats and Republicans to one of two conditions: own-party or other-party. In the *own-party condition*, participants rated their party’s reasons for engaging in conflict with the opposition party, asking them how motivated their party is by love for their own party versus hate for the other party. In the *other-party condition*, participants rated the opposing party's reasons. Results showed that Democrats and Republicans believed that their own party was primarily motivated by ingroup love, whereas the outparty was motivated by outgroup hate. In a follow-up experiment, Waytz and colleagues randomly assigned participants to an incentive or control condition. In both conditions, participants were told that they would soon rate outgroup motives and that their responses would be compared to the actual responses of people in the opposing party. Those in the *incentive condition* were told that they could earn a bonus payment of $12 for accurately gauging the opposing party’s motivations. Results demonstrated that those in the incentive condition became more likely to rate the outparty as motivated by ingroup love. This change in perceived motives for outgroup actions was, in turn, associated with more favorable views of the outparty’s morality (i.e., reduced blame).

In short, a diverse set of interventions appears to reduce partisan blame by revealing that outparty members adopt their morally disagreeable beliefs and take their morally wrong actions for understandable, compelling reasons.

#### Interventions that Target Freedom of Action

To the best of our knowledge, the only exploration of the impact of freedom of action perceptions on partisan animosity is Severson’s (2020) unpublished manuscript. Severson randomly assigned participants to either a biological treatment group or a control group. In the biological treatment group, participants were exposed to an article summarizing research on the neurobiological and genetic underpinnings of political beliefs, while those in the control group read about sloths. The findings revealed that participants in the treatment group were less inclined to believe that political ideology was solely a matter of free choice, leaning more toward seeing a role of biological determinism. The results also showed that participants who strongly believed that ideology was a matter of free choice tended to hold more negative feelings toward political outgroups. Finally, there was an indirect effect of the intervention on negative feelings toward the outparty: Democrats and Republicans who came to see an impact of biological determinism on political ideology showed an associated tendency toward reduced blame of the outparty. This depolarizing effect was strongest among highly partisan individuals.

#### Interventions that Target Control of Self-formation

Research has shown that diminishment of perceived control of self-formation tempers partisan animosity. In one experiment, Gill et al. (2023) manipulated whether partisans learned the formative history of a political opponent (i.e., how she developed her political beliefs under the influence of family, community, and religion) or learned non-explanatory, neutral information about her (i.e., she’s from Denver, hates the cold winters). Then, participants read a harsh tweet she sent in which she criticized the participants’ political beliefs. Participants had the opportunity to tweet a reply, and this reply was coded for hostility. Results showed that when liberals and conservatives learned about the political upbringing of the harsh tweeter, their replies to her became less hostile. This effect was mediated by viewing the tweeter as less in control of developing her beliefs (i.e., reduced control of self-formation), and, therefore, less blameworthy. In follow-up experiments, Gill and colleagues replaced the individual narrative about the harsh tweeter and instead gave participants a *historicist reminder—*a message about how, as a general fact, formative experiences shape *everyone’s* political beliefs. They discovered that this general historicist reminder reduced the harshness of tweet responses among liberals but not conservatives, and this effect (for liberals) was, again, mediated by reductions in perceived control of self-formation and blame.

In a more recent paper, Alam and Gill (2024) extended this work to the context of changing attitudes toward the outparty as a whole (i.e., Democrats, Republicans). In their experiments, they randomly assigned partisans to a historicist thinking intervention or control. The intervention consisted of three elements: (a) a narrative detailing the development of a particular political opponent’s worldview (as in Gill et al., 2023), coupled with (b) a message emphasizing how individual life experiences influence *everyone’s* political beliefs (as in Gill et al., 2023), and (c) a suggestion that outparty members can change through future formative experiences. They found that for both Democrats and Republicans, the intervention reduced animosity toward the outparty as measured by the feeling thermometer. In follow-up experiments that sought to identify the particular blame emotions affected by their intervention, they found that for Democrats the intervention increased compassion for Republicans, while for Republicans, it reduced disgust, disapproval, and anger toward Democrats. For Democrats, the effects of the intervention were mediated by decreased perceptions of Republican control of self-formation across all experiments. For Republicans, however, the effects of the intervention on reduced animosity, while present, were not mediated by reductions in perceptions of control of self-formation.

#### Interventions That Target Dispositional Attributions

Other interventions have arguably targeted dispositional attributions. They do so by providing information to correct people’s negatively skewed views of outparty character. Such interventions are inspired by the fact that political partisans often hold exaggerated views of the immoral character of the opposing party based on inaccurate perceptions regarding the prevalence of extreme and objectionable beliefs among outparty members. For example, Democrats and Republicans overestimate the extent to which outparty members hold anti-democratic attitudes and are willing to use violence for political purposes (Mernyk et al., 2022; Pasek et al., 2022). Similarly, partisans overestimate how much outparty members dehumanize and dislike them (Landry et al., 2023; Moore-Berg et al., 2020). Together, these misperceptions give rise to negative dispositional attributions regarding the outparty: They are violent, callous, mean, and so on. In a truly vicious circle, these exaggerated perceptions of outparty wickedness predict reciprocal *inparty* animosity toward the outparty (e.g., Landry et al., 2023; Mernyk et al., 2022; Moore-Berg et al., 2020).

Many interventions aim to improve beliefs regarding outparty dispositions by correcting these underlying exaggerations and misperceptions. For example, Landry et al.’s (2023) intervention corrected exaggerated perceptions of meta-dehumanization by exposing participants to information on the actual (relatively low) extent to which the outparty dehumanizes the inparty. This correction reduced hostility toward outpartisans, an effect that remained evident one week later. In Voelkel et al. (2024) mega-study of 25 interventions to reduce partisan animosity, they included five interventions that corrected different types of misperceptions. These misperceptions included beliefs about the willingness of outpartisans to break democratic norms and to sacrifice lives to the pandemic for political gains. All five interventions significantly reduced partisan animosity, arguably by reducing negative dispositional attributions regarding the outparty’s supposed unfairness, heartlessness, Machiavellianism, and so on.

Moreover, the intervention with the largest effect size tested by Voelkel et al. (*d* = −0.53; 2024) was a video that featured pairs of opposing partisans discussing their political differences. The video featured three pairs of partisans, a leftist feminist and a rightwing antifeminist; an environmental activist and a climate change denier; and a transgender woman and a man who believed gender is binary. Prior to learning about their partner’s political views, the pairs did a variety of “ice breaker” and “team building” tasks with their partners. After these shared activities, the pairs viewed recordings of earlier interviews where their partner expressed their opposing political opinions. After this, they were offered the choice to discuss their differences over beer or to leave. All three pairs chose to stay and cordially discuss. Although it was not directly measured, and many variables were presumably manipulated by the video, we would wager that this intervention was effective at reducing partisan animosity, in part, because it reduced negative dispositional attributions regarding the outparty. By highlighting the presence of likeable outpartisans—people who were friendly and cooperative during the “ice breaker” tasks, people who were open-minded and willing to cheerfully chat over beer even after learning of deep political differences with their partners—the intervention challenged perceptions of outpartisans as dispositionally nasty and closed-minded.

Researchers have also examined a variety of interventions that teach individuals how to communicate constructively with outpartisans. We suggest that these interventions, too, are effective because they remove blame-aggravating dispositional attributions regarding the outparty. That is, those who learn to communicate positively—respectfully, open-mindedly—are seen as having positive dispositions, thereby reducing outpartisans’ animosity toward the communicator and her inparty. For example, Minson et al. (2024) trained liberals and conservatives to craft political persuasion messages for outpartisans that expressed “conversational receptiveness”, or a willingness to thoughtfully engage with opposing views. Those trained in this communication style received more positive evaluations from an outparty member and, more broadly, improved that outparty member’s view of the communicator’s inparty. Similarly, Puryear and Gray (2024) instructed partisans to use “balanced pragmatism”—considering multiple perspectives and seeking pragmatic solutions—when writing about their views on immigration. Compared to communicators instructed to focus on being logical and cohesive, those pursuing balanced pragmatism were judged by outparty members as more respectful and as more desirable as an interaction partner. This effect was mediated by perceiving these balanced pragmatists as being more rational and moral. Finally, Collins et al. (2022) found that partisans tend to underestimate how willing outpartisans are to learn about opposing views. When participants were informed that their ideological counterparts were indeed interested in understanding opposing viewpoints, they reported more favorable evaluations of outpartisans and expressed a greater willingness to engage with them in the future. By enabling partisans to demonstrate positive dispositions via positive communication strategies, these interventions reduced partisan animosity (see Chen et al., 2010; Hussein & Tormala 2021, 2024; Kalla & Broockman, 2023; Minson & Chen, 2022; Yeomans et al., 2020; for similar findings).

#### Interventions that Target Identity-Based Biases in Blaming

Above, we described how social identity motives can motivate overblaming of the outparty. One way to overcome such identity-based biases is to alter partisans’ sense that they belong to two discrete, mutually exclusive groups (i.e., reduce the “sharp sense of us-vs-them” described above). Indeed, researchers have found that highlighting commonalities between the parties can reduce partisan animosity. Wojcieszak and Warner (2020) had Democrats and Republicans engage in vicarious or imagined positive contact with the outparty. They found that both forms of contact reduced partisan animosity and did so by increasing perceived commonality—that is, shared identity—between oneself and the political outgroup. Similarly, Ahler and Sood (2018) corrected inaccurate perceptions of outparty demographic composition (e.g., percentage of Democrats who are part of the LGBTQ community; percentage of Republicans who are extremely wealthy) and found that this reduced partisan animosity. Though not explicitly measured, it seems likely that their intervention—by reducing how demographically different the outparty seemed—increased perceived commonality between the two parties. Finally, Voelkel et al.’s (2024) intervention tournament found that interventions that highlighted commonalities between Democrats and Republicans, such as their shared exhaustion from excessive political conflict or their common national identity as Americans, tended to be particularly effective for reducing animosity.

In short, increasing perceived commonality between political parties—challenging the idea that they are discrete, mutually exclusive entities—arguably reduces the social identity-based motivation to heap blame on the outparty, and thereby reduces partisan animosity.

### Interventions That Target Determinants of Blame Expression

#### Interventions That Target Beliefs About the Efficacy of Blame Expression

Some successful interventions appear to target beliefs about the efficacy of blame expression. Santos et al. (2022) tested the impact of increasing participants’ belief in the utility of having empathy for political opponents. In an experiment, they randomly assigned participants to one of two conditions: A high-utility condition where they read about how cross-partisan empathy can increase an individual’s political persuasiveness (e.g., *treat your opponent with respect, and then they are more likely to compromise*), or a low-utility condition where they read about how cross-partisan empathy can decrease an individual’s political persuasiveness (e.g., *you will appear weak if you don’t stand up for your beliefs*). The results showed an impact on blame expression: Participants in the high-empathy-utility condition utilized more conciliatory language when communicating with their political opponents, which, in turn, led to their opponents liking them more and judging their messages to be more persuasive. In short, when people are reminded that hostile blame expression is inefficacious—empathic communication is superior—their cross-partisan communication becomes kinder and partisan animosity is thereby reduced.

#### Interventions That Target Normative Beliefs About Blame Expression

Lastly, other successful interventions to reduce partisan animosity have targeted beliefs about the normative appropriateness of blame expression. For instance, Munger (2021) conducted a field experiment on social media, employing Twitter (now X) accounts to sanction individuals for publicly expressing harsh blame toward outpartisans. Initially, Munger created Twitter accounts in 2015 to simulate Democrat and Republican users. Subsequently, he scraped Twitter for hostile tweets from non-elite partisans directed at Donald Trump or Hillary Clinton prior to the 2016 U.S. presidential election. These hostile partisans were then randomly assigned sanction messages from in-party accounts created by Munger (e.g., Republicans who harshly tweeted at Hillary Clinton were sanctioned by one of Munger’s Republican accounts). Both Democrat and Republican users were assigned one of three sanctions: Care-based moral argument (“You shouldn’t use language like that . . . our opponents are real people with real feelings”), authority-based moral argument (“You shouldn’t use language like that. . .[we] need to behave according to proper rules of political civility”), or a non-moral public sanction (“Everyone can see that you tweeted this.”). Another group of participants received no sanctions. Results revealed that the sanctions with a moral basis (i.e., care- or authority-based) reduced political incivility across the first week post-treatment. That is, participants sent fewer uncivil tweets during the week after being sanctioned by morally grounded messages. We view Munger’s successful messages as signaling to partisans the normative inappropriateness of nasty blame expression, thereby reducing their willingness to publicly express partisan hostility.

### Summing Up

In short, a wide variety of existing, successful interventions to reduce partisan animosity appear to derive their efficacy from the fact that they target one or more of the variables that govern blame attitudes and blame expression (see Figure 3).

## Depolarizing the Future: Possible Interventions to Reduce Partisan Animosity Inspired by Our Blame Framework

The preceding section showed that the psychology of blame (see Figure 3, especially) provides a unifying framework for understanding existing successful interventions to reduce partisan animosity. Of course, a strong conceptualization of a phenomenon should also be generative, stimulating promising new research ideas regarding the phenomenon of interest. Below, we aim to establish the generativity of our blame framework by proposing a diverse array of novel interventions based on that framework. The interventions we propose below are grounded in the same principle as those we reviewed earlier: They aim to reduce animosity by prompting reappraisal of already-existing intense attitudes of blame or by prompting down-regulation of the expression of such attitudes (i.e., they assume that partisan animosity is already at a high level). Typically, they prompt reappraisal through exposure to new information about the outparty. They prompt downregulation by exposure to new information about appropriate or effective communication tactics. We do not endeavor to design an intervention connected to each blame concept in Figure 3. Rather, we will pick and choose with a focus on interventions that strike us as relatively novel and promising. We hope that other researchers will be inspired to use their creativity and insight to come up with even more possible interventions.

In designing interventions, it would be worthwhile to keep in mind that blame reduction in a partisan context is likely to be particularly challenging (see our discussion of bias in relation to Figure 2). Indeed, Gill and his colleagues, for example, have found large blame-mitigating effects of historicist narratives in non-political contexts (e.g., average *d* = −0.94 in Gill & Cerce, 2017) which become quite a bit smaller when imported into political contexts (e.g., average *d* = −0.35 in Gill et al., 2023). Because of such challenges, researchers should certainly consider interventions that combine various types of blame-mitigating strategies, such as, say, weakening partisan identities prior to presenting blame-mitigating information about the outparty.

### Offense Severity

One strategy to diminish partisan animosity could involve reducing exaggerated beliefs about the harm caused by outparty’s policies. Above, we described existing interventions that reduced animosity by correcting exaggerated perceptions of negative outparty dispositions. Here, the intervention focuses instead on correcting exaggerated perceptions of consequences of outparty actions. For instance, conservatives might hold inflated perceptions regarding the impact of gun regulations on crime rates, believing that such measures lead to a significant increase in robberies, home invasions, and murders of innocent family members. An intervention could start by prompting conservatives to give their perceptions regarding the effects of gun regulations on crime rates. Subsequently, the intervention could present data on the actual trends in crime rates—ideally presented as part of an “adversarial collaboration” including both liberal and conservative scholars—following the implementation of these regulations. By demonstrating that the perceived increase in crime is not supported by empirical evidence (Hemenway, 2011; McDowall et al., 1991), the intervention could correct exaggerated beliefs about harm, which should reduce the perceived offense severity of outparty policies and thereby dampen partisan overblaming.

Another approach to reducing perceptions of offense severity could be to enhance awareness of how politicians are incentivized to exaggerate offense severity to gain citizens’ attention or loyalty. This could be done, for example, by sharing the research we reviewed above about politicians becoming more uncivil on Twitter (Frimer et al., 2023) due to the incentives—i.e., virality—associated with incivility (Brady et al., 2021). It could also be done by sharing the research we reviewed above on how political elites pursue their own self-interest by exaggerating the supposed harms created by a scapegoat group (Banda & Cluverius, 2018; McCoy & Somer, 2019). By educating the public on the perverse incentive structure of social media and on the self-interested motives of political elites, partisans might become more likely to take with a grain of salt any political rhetoric regarding the supposed “outrageous harms” that arise from outparty policies. Consequently, this informed skepticism—or enhanced “bullshit detection” (Littrell & Fugelsgang, 2024)—might lead people to resist adopting politicians' exaggerated narratives of offense severity, thereby reducing partisan animosity.

Yet another approach could focus on the role of language in shaping perceptions of offense severity. It is a truism that politicians strategically use language to manipulate perceptions of offense severity. For example, although both labels might arguably be fitting, calling an opponent a *hatemonger* implies that she causes much more harm than if she is labeled a *provocateur* (Walker et al., 2021). We have no interest in endorsing the practice of manipulative language use, so we propose an intervention to reduce perceived offense severity by using bland factual information, which is, to the extent possible, stripped of any manipulative use of language (e.g., *Disastrous! Worst ever! Complete failure!*). One promising intervention could involve first quoting a political speech that used manipulative language to create exaggerated perceptions of offense severity: “Voters will not forget or forgive all the misery and despair Crooked Joe Biden has caused in just four years. . . . After almost four years of Biden’s disastrous presidency, we need a return to America First policies that successfully. . .supercharged the economy for all Americans” (Trump spokesperson Steven Cheung, quoted in Politico, 2/2/24). This hyperbolic portrait of President Biden’s negative impacts could be juxtaposed with a more neutral, factual portrait. To promote a sense of legitimacy for the factual information, it could be delivered by a team of economists who differ in their political allegiances (for a nice example of this approach, see Francis, 2012). We are not economic experts, and we would defer to the judgment of such experts in terms of what information to present and how to frame it as neutrally as possible. We suggest, however, that the economists might point out that—under President Biden—the United States avoided a recession that was strongly predicted for 2023 (Zinkula, 2023), and job growth exceeded expectations for several years (U.S. Department of Commerce, 2024). Of course, the economists should also point out that inflation remained a problem in 2024 and was arguably caused by some of President Biden’s COVID stimulus policies (de Soyres et al., 2022). They could also note, however, that Americans differ in their views of how much of a problem it is (Pew Research Center, 2024). Furthermore, to counter the misleading implications of former President Trump’s spokesperson, the bipartisan team of economists could point out that President Trump’s policies led to a large increase in U.S. National Debt (Congressional Budget Office, 2020). Finally, they might also point out that it is difficult to assess the wisdom of President Trump’s economic policies during his first term because a global pandemic created substantial disruptions to the U.S. economy during that time. Obviously, this flurry of factual information does not fit with any simple-minded narrative about how outparty policies “destroy everything” and thus it should weaken feelings of animosity toward opponents.

### Intentionality and Reasons

Another potential avenue for future interventions involves reshaping perceptions of the motives and intentions that guide members of opposing political parties. As mentioned earlier, perceived intentionality—a powerful contributor to blame—is related to viewing agents as both *knowing/foreseeing* and *desiring* the consequences of their actions. Consequently, altering such perceptions holds promise in diminishing partisan animosity. For instance, Democrats may form an intense attitude of blame toward Republicans who supported a Pro-Life candidate who subsequently changed laws to require victims of rape to carry the child of their rapist. In such cases, however, Republican voters may have been unaware of the candidate's intent to enact such a change, and they might not have desired such an outcome. In fact, most Republicans believe that abortion should be legal in cases where a pregnancy results from rape (Hartig, 2022). One can imagine many instances like this, where the anticipated and desired outcomes of partisans differ from actual political changes. An intervention that lowers the perceived extent to which outparty voters could or did foresee a particular noxious political outcome could be an effective way to reduce partisan animosity.

Another type of intervention could focus on the fact that people often adopt political beliefs and join political parties based on intentions and reasons that have little to do with the beliefs or parties they ultimately embrace. For example, Munson (2010) has shown that people often join political movements out of a desire to be part of a community and to maintain friendships. Thus, a person might attend a political rally based on an invitation from a friend or colleague, and not because she has strong feelings about the focal issues of the rally. Over time, however, with continued attendance at similar rallies, she will start to care—or perhaps pretend to care—about those focal issues based on an intention to be a good member of a community that she joined purely based on happenstance. Here, an intervention that highlights how non-political intentions (e.g., being a good friend, being a good community member) can motivate outpartisan political actions might be another way to reduce partisan animosity. It’s hard to despise people who are simply trying to connect with others in their community.

### Freedom of Action

Another way to reduce partisan animosity might involve changing perceptions of outpartisan freedom of action. For example, future research could extend Nichols and Knobe’s (2007) deterministic universe manipulation or Shariff et al.s’ (2014) neuroscientific manipulations of freedom of action in the context of politics. That is, after presenting people with descriptions of a deterministic universe or neuroscientific arguments against free will, researchers could measure partisan animosity. Another approach could involve having participants learn about the neuroscience of pre-conscious decision-making. For instance, they could be introduced to studies showing that when individuals make simple decisions (e.g., pressing a button to choose between two options), brain scans reveal neural activity that predicts their choice *before* they become consciously aware of making it (Soon et al., 2008). This intervention could be enhanced further by having participants engage in these activities themselves. For example, participants could enter an fMRI scanner, make a series of simple decisions, and then be shown neural activity that “predicted” their choices before they became consciously aware of making them. Experiencing this firsthand could make the intervention more personalized, and therefore more effective and durable. Of course, such an approach would be quite resource-intensive. The goal would be to diminish people’s belief in freedom of action with respect to political decision-making or perhaps even their general belief about whether people ever have freedom of action at all. For these interventions to influence political animosity, however, it is likely important to identify ways to connect these abstract ideas about free will and determinism to political beliefs specifically. One way to do this would be to, say, make the fMRI-based intervention about political judgments, such as choosing between pairs of candidates and policies. Embedding such framing directly within the intervention would help participants generalize arguments for determinism to the political domain, making the exercise more directly relevant to partisan blame. Our predictions are that such manipulations could reduce partisan animosity by encouraging partisans to view individuals, including outparty members, as lacking the freedom to act otherwise (i.e., because choices are driven by neural activity that precedes awareness that one is making a choice, outpartisans were not free to vote another way at that time or to choose to feel differently about the issues that are most important to them).

Above, we noted that perceived freedom of action can also depend on perceived freedom from external constraints on one’s decision-making. In the political realm, there are scenarios where choices are constrained due to external constraints such as social pressure. For example, in the context of religious beliefs, the Associated Press (Fam, 2023) has documented how atheists in the Middle East and North Africa hide their non-Islamic views given their fears of being ostracized by members of their predominantly Muslim societies, including their own families. A similar dynamic may apply to American political beliefs and party affiliation. For example, Gibson and Sutherland (2023) have found that 46% of Americans do not feel free to express their views, a percentage that has tripled since 1954. This means that, for example, a young woman in liberal Massachusetts might be reluctant to fully embrace an anti-abortion stance—even though such a stance accords with her moral intuitions—because doing so feels too risky. What if her liberal family, friends, and neighbors found out? She would be an outcast. Indeed, Gibson and Sutherland show that self-censorship can be driven by fears of being ostracized by friends and family. Relatedly, Matthes et al.s’ (2018) meta-analysis of research on the “spiral of silence” reveals that people are least likely to express their dissenting views to their close social circles, such as friends and family. Here, an intervention could make salient to partisans the costs that outparty members face for deviating from their party, such as being socially isolated or ridiculed. Such an intervention could also be paired with information on the damaging mental and physical health consequences of ostracism (e.g., Holt-Lunstad et al., 2017; Office of the Assistant Secretary for Health, 2023). It would become harder to hate outparty members if one recognizes that many of them toe the line due to fear of ostracism rather than due to a freely chosen and sincere embrace of outparty ideology.

### Control of self-formation

Though prior work has already applied historicist thinking interventions to reduce partisan animosity by diminishing perceptions of control of self-formation (Alam & Gill, 2024; Gill et al., 2023), these interventions could be strengthened further with the goal of producing larger effect sizes of more practical importance. In Hartman and colleagues’ review of partisan animosity interventions (2022), they discuss how interventions may need to involve “experiential learning and personal relevance” to become durable. Most research on historicist thinking, in contrast, has employed very brief vignette-style narratives that participants were tasked to read. A more powerful intervention in this context may require having partisans have face-to-face conversations in which they discuss their backgrounds and how such backgrounds have shaped their political beliefs. Another approach could be to employ virtual reality methods (Taylor et al., 2020). For example, a VR simulation could involve partisans experiencing a fast-forwarded, typical upbringing of an outparty member. Such a simulation could highlight the influence of parents, religious institutions, media environments, and other powerful formative influences to help partisans understand how opposing political beliefs are sometimes formed. Finally, another approach might be to create documentary films regarding the political formation of members of different political parties. These richer, more experiential interventions might lead to larger effect sizes and longer-lasting effects than those achieved by the brief vignette-based interventions in the existing literature.

### Dispositional Attributions

Challenging the validity of negative dispositional attributions regarding the outparty can be an especially fruitful way to reduce partisan animosity. One way to do this could be to highlight the irrationality or unfairness of inferring negative group dispositions from the behavior of a small number of group members. As noted above, Bruneau et al. (2018) accomplished this by having White Americans reflect on how blameworthy they and their racial group are for White supremacist terrorist attacks. After such reflection (*Of course, I have nothing to do with—and most White people have nothing to do with—Dylan Roof’s murder of Black parishioners!*), White Americans assigned far less collective blame to Muslims as a group for Islamist terrorist attacks. A similar intervention could be applied in the context of other groups. Partisans may be blaming the outparty as a whole for individual (or small group) acts of political violence, hate speech, and more, but not doing the same for their inparty. For example, in the American context, Republicans may be blaming Democrats as a group for Hillary Clinton calling Republicans a “basket of deplorables,” yet they may not feel responsible for the incendiary statements made by Donald Trump. By having partisans reflect on examples of these double standards, and thereby creating cognitive dissonance (Festinger, 1957), partisans may be less prone to inferring negative dispositional attributions regarding the outparty as a whole for individual (or small group) offenses (*Not all Democrats believe we are a “basket of deplorables”*). Naturally, this should reduce partisan animosity.

Another way to change perceptions of the outparty’s ostensibly collective dispositions might be an intervention that involves unpacking political parties into subgroups. Halevy et al. (2022) had Israelis allocate blame to various groups and subgroups for the absence of a solution to the Israel-Palestine conflict. They assigned participants to one of two conditions: Israeli side unpacked or Palestinian side unpacked. In the Israeli side unpacked condition, participants were asked to allocate blame among the Israeli right-wing bloc, the Israeli center bloc, the Israeli left-wing bloc, and the Palestinians. In the Palestinian side unpacked condition, participants were asked to allocate blame among the Palestinian Hamas movement, the Palestinian Fatah movement, the Palestinian Islamic jihad movement, and Israel. They found a significant shift in blame judgments by condition. When the Israeli side was unpacked into different subgroups, rather than just as the nation of Israel, much more blame was assigned to the Israeli side than to the Palestinian side, with this effect driven by very high amounts of blame assigned to the Israeli-right-wing bloc (and not much assigned to other Israelis). This intervention may have prompted Israelis to consider the negative traits associated with this bloc (e.g., callous attitudes toward Palestinians) that have exacerbated the conflict, leading to increased blame of them (and not of Israelis as a whole) for the intractability of the conflict.

Partisan animosity in other nations might be disrupted by a similar process. In the American context, unpacking Democrats and Republicans into subgroups, such as congressional legislators vs. radical-fringe groups vs. “average Joes/Janes,” and so on, could help particularize blame toward specific actors by making obvious the fact that only particular subgroups have objectionable dispositions (e.g., unfair, anti-Democratic, etc.). By highlighting specific actors or subgroups that are most blameworthy, this could reduce the amount of animosity partisans feel toward the outparty as a whole. For example, in the context of January 6th Insurrection, unpacking Republicans into subgroups could influence Democrats to assign greater blame toward Donald Trump and the rioters than toward ordinary Republicans, hence reducing partisan animosity held toward most Republicans. Similarly, Republicans could be nudged to blame fringe protesters for rioting and looting during Black Lives Matter (BLM) protests, instead of blaming Democrats as a whole. Indeed, one can imagine many cases in which subgroups—or even small numbers of individuals—may be seen as more blameworthy for political conflict than Democrats and Republicans as collective entities. By encouraging partisans to consider the role of specific subgroups in fueling political division, they might then attribute more positive dispositions to the outparty as a whole, reducing partisan animosity.

Both interventions described above—collective blame hypocrisy; “unpacking” into subgroups—involve undermining perceptions of *group entitativity*. That is, the interventions prompt recognition that there is no singular, unified entity called *Muslims* or *Israelis* or *Republicans* or *Democrats* that, acting in concert, has done wrong *en masse*. The interventions do this by particularizing blame, directing it toward particular individuals or subgroups within a larger ideological group. Although prior research on perceived group entitativity has not explored blame per se, the research does suggest that the formation of group-directed attitudes (e.g., stereotypes, prejudice) is facilitated by perceived group entitativity (Hamilton et al., 2002). Thus, because partisan blame is a group-directed attitude, perceived entitativity of ideological groups should facilitate partisan blame. We encourage researchers to focus on novel and creative interventions for undermining the perceived entitativity of ideological groups with interventions that operate via mechanisms other than particularizing blame. One possibility in the American context might be to create, say, a series of TikTok videos in which members of a political party state one point on which they agree with their party and one point on which they disagree. For example, across a couple dozen short videos, viewers might hear one registered Democrat say “I agree that health care for all should our goal” but “I am not a fan of the ‘wokeness’ that is common among other Democrats,” and then another registered Democrat might say “I agree that we need a more generous social safety net” but “I disagree with the idea that we should have totally open borders—we need tighter control at the border,” and so on. After seeing many such videos, “Democrats” will come to seem more like an aggregate of distinct individuals than a singular, unified collective entity. Because it makes little sense to despise a collective entity that has been shown to lack real existence, this intervention should reduce partisan animosity.

Interestingly, these interventions targeting negative dispositional attributions might also reduce perceived *ideological* polarization (i.e., in addition to reducing *affective* polarization). That is, by prompting partisans to reconsider negative images of the outparty—images often based on a few individuals or subgroups—they may come to see that the outparty is not as extreme or different from them in its positions as they initially believed.

This dynamic of blaming the outparty as a whole for the beliefs and actions of a few of its members may be especially pronounced in two-party systems, such as the United States, where the presence of a single outparty fosters sweeping group-directed blame. By contrast, in multiparty systems, citizens face numerous outparties, which can dilute broad group-directed blame and instead channel hostility toward those factions seen as most responsible for political ills. Wagner’s (2021) cross-national study of affective polarization supports this view, showing that animosity in multiparty contexts often clusters against specific radical or extreme parties rather than being directed uniformly at all non-inparty members. Therefore, multiparty systems may naturally encourage more particularized blame and less animosity between particular pairs of ideological foes.

Group malleability interventions—interventions highlighting how groups can change because their conditions or leadership change—could also reduce partisan animosity. With this approach, the idea is that the outparty’s negative dispositions can *change* rather than that they do not exist. For example, Halperin, Russell, Trzesniewski, et al. (2011) had Israeli and Palestinian participants read a fictitious article describing groups that had committed violence (without explicitly mentioning Israelis or Palestinians) and randomly assigned them to either a malleable or fixed condition. In the malleable condition, the article suggested that the groups had changed their behavior over time because their violence was a temporary outburst caused by external factors like corrupt leadership or a particularly oppressive environment. In the fixed condition, the article suggested that the groups had not changed, and their violence was rooted in inherent and durable group characteristics. Participants in the malleability condition developed significantly more positive attitudes toward the outgroup and expressed a greater willingness to compromise and make concessions about the conflict. Extending this research, Goldenberg et al. (2018) demonstrated that such interventions, in the form of workshops, can have long-lasting effects in reducing negative attitudes toward the outgroup, fostering hope for peace, and increasing willingness to make concessions, even during periods of intense violence. A similar intervention could be applied in the context of American partisan animosity. By having partisans engage with examples of how their own party or group has changed over time, such as shifts in positions on civil rights and other social issues, they may be less inclined to view the outparty as incapable of change. Such an intervention could reduce attribution of *durable* negative dispositions to the outparty and subsequently diminish partisan animosity.

### Counterfactual Thinking

Another novel intervention could undermine partisans’ blame-aggravating counterfactuals. For example, in the United States, partisans on both sides of the ideological divide believe the other side is to blame for increased polarization following the COVID pandemic. Each side imagines—counterfactually—that *if only* the outparty had been more ethical and intelligent in handling the crisis, subsequent political polarization *would have* been avoided (e.g., *If only Trump and his followers would have trusted the experts!; If only the liberals would have opened schools and businesses sooner!).* One possible intervention would undermine these counterfactuals by presenting (factual) data from a cross-national survey revealing increased division and polarization *in most countries* post-COVID (Silver, 2022). If division and polarization have increased all around the globe, then it is very likely that merely undoing outparty’s actions at home would undo division and polarization at home. By confronting individuals with factual data that contradicts their counterfactual assumptions, this type of intervention could reduce partisan animosity.

### Down-Regulation of Blame Expression

Other interventions could focus on motivating down-regulation of harsh blame expression toward outpartisans. One approach could highlight the inefficacy of harsh blame expression. For example, Gill et al.’s (2025) in-person workshops that highlighted the damaging consequences of harsh blaming in relationships could be exported to this context. Some research has examined such negative consequences specifically in the context of political polarization. As one example, Velez and Liu (2024) showed that harsh criticism of a person’s belief causes a “backfire effect” in which the person becomes even more extreme in their initial belief. Velez and Liu had participants provide descriptions of their deeply held political views, which were used to generate personalized counterarguments using ChatGPT. Across a series of experiments, participants were randomly assigned to receive counterarguments varying in tone, from neutral and respectful to hostile and vitriolic. On a positive note, exposure to neutral and respectful counterarguments often led participants to question their own position (i.e., they were open-minded!). In contrast, however, exposure to hostile, blaming counterarguments produced the backfire effect mentioned above. Because, naturally, no one wants to increase the extremity of outpartisans’ beliefs, teaching partisans about this backfire effect caused by harsh blame expression could reduce public expressions of partisan animosity.

Moreover, it is possible that partisans may believe that expressing harsh blame toward outpartisans is appropriate and a norm within their political ingroup. Challenging such perceived norms could be another effective intervention. Indeed, this norm perception is, in fact, false. Research by Heltzel and Laurin (2021; also see Druckman et al., 2019; Frimer & Skitka, 2018) suggests that, in reality, partisans admire in-party members who listen and try to understand the views of their political opponents, as they view such inpartisans as tolerant, cooperative, and rational. However, they also found that this effect weakened, though did not disappear, when inpartisans engaged with politically extreme outpartisan beliefs, especially when they were perceived as validating or even adopting such beliefs (also see Hussein & Wheeler, 2024). Given this latter finding, it is important to recognize that partisans tend to perceive outparty beliefs as more extreme than they actually are (Mernyk et al., 2022; Pasek et al., 2022; Yudkin et al., 2019). When they have such exaggerated perceptions of outparty extremity, then describing in-party members as seeking out opposing perspectives might be a relatively ineffective intervention because partisans may wrongly assume that in-party members are seeking out outparty members with profoundly unacceptable views. Therefore, an intervention that first corrects misperceptions about the extremity of outparty beliefs, followed by an intervention that challenges the belief that hostile rejection of the outparty is an ingroup norm (e.g., by providing data showing that inparty members actually disapprove of such behavior and admire open-minded individuals), could be particularly effective in reducing outward expressions of partisan animosity.

Finally, another norm-based intervention could leverage eudaimonic motives for blame regulation. For instance, partisans could reflect on whether publicly expressing anger and hostility aligns with their conception of leading a meaningful and moral life. One approach could involve asking participants to think of a person they deeply admire for their moral character and whose values they aspire to embody—such as a historical figure, mentor, friend, or even a fictional character. They could then imagine how this role model might respond to political disagreements. Would this person engage in hostility toward outpartisans, or would they try to demonstrate compassion and humility? Presumably, most participants would envision someone who chooses the latter. Encouraging partisans to reflect upon how public expressions of partisan animosity contradict their ideal of “being a good person” could be a fruitful avenue for reducing outward expressions of partisan overblaming.

Again, although our focus is on reducing partisan overblaming, it is interesting to note that interventions to encourage self-regulation of blame expression could also reduce both perceived and actual *ideological* (in addition to *affective*) polarization. By encouraging partisans to engage in more constructive communication with outpartisans, actual conversations might become more likely. This could lead to increased awareness of points of agreement, thereby reducing perceived ideological polarization. Moreover, exposure to respectful outparty communicators could also make people more open to considering outparty views, potentially even adopting some of them, which would reduce actual ideological polarization.

## Our Positionality, Constraints on Generality, and Citation Statement

In the aftermath of hearing our perspective on the dangers of partisan animosity and the importance of reducing it, the reader should know a bit about our social and educational backgrounds, which have undoubtedly affected the development of our thinking, worldviews, and methodological perspective. Interestingly, our backgrounds are in some ways quite different—the first author is a first-generation American born to South Asian Muslim parents and raised in a liberal West Coast city, the second author is a descendant of Irish and Italian Catholics who emigrated to America around the turn of the 20th century and he has lived both in the liberal northeast and in the conservative southeast U.S.—yet we have very similar views regarding the issues that we study. Both authors are American, and their collaboration began in a university located in a purple/swing political state and city, characterized by both conservative and liberal ideologies. Moreover, both authors have grown up with friends and family with different political beliefs. Since high school, the first author has hesitated to discuss certain religious beliefs with his family due to concerns about potential conflict and ostracism. The second author has seen firsthand how disagreements over morally significant political issues can sever family bonds, but he has also seen how people can have a relationship of love and respect despite staunch disagreement over such issues. He is inclined to think of blame as a bad thing (especially when it is overdone), but both he and the first author regularly engage with the ideas of others who emphasize the social benefits of blame. The first author is receiving doctoral training in social psychology at a public university and currently researches moral disagreement, punishment, and collective action. The second author received doctoral training in personality and social psychology and currently researches the psychology of blame and punishment at a private university in the northeastern United States where he has taught and researched for 27 years.

Perhaps more importantly, the reader should understand some of the broader assumptions we make. Indeed, much work in social psychology is based on sometimes unstated assumptions about what a “better world” would look like. We believe that the development of a pluralistic, diverse world in which differences among citizens do not lead to intense and destructive hostility is ideal. The absence of intense and destructive hostility does not mean the eradication of ideological diversity. Rather, it means that those with divergent points of view can engage in respectful interactions, seek common ground, compromise, persuade, offer mutual respect, become friends, and so on. This notion of an ideal society is a motivating force that pushes us to uncover empirically grounded solutions to the problem of overblaming in the political realm and beyond. Our assumption is that reducing blame is crucial and that this can be done even if citizens continue to view outparty beliefs and actions as the wrong ones for the nation.

With respect to our disciplinary and methodological backgrounds, we are experimental social psychologists and we draw primarily from research in that subfield. We believe that to intervene in a way that changes the world, one must understand the causes of the thing one is trying to change. This is why we rely heavily on insights gained from experimental work, because such work is uniquely well-suited to identifying cause-and-effect relations. At the same time, we acknowledge that the methods in our field often involve artificial and contrived testing conditions with a focus on short-term changes in cognition or emotion. Thus, it is crucial that cause-and-effect relations found in the lab are tested in more realistic contexts and with respect to more durable impacts on people’s thoughts and feelings (for great examples see Binning et al., 2024; Fishbane et al., 2020; Gerend & Shepard, 2012; Mortensen & Cialdini, 2010; Plant & Peruche, 2005). Indeed, some of our proposed future directions in our article explicitly call for such research.

We also recognize that much of the research we cite was conducted primarily by North American and European scholars on WEIRD participants (Henrich et al., 2010). This does not reflect a deliberate choice on our part but rather reflects the broader pattern that most studies on affective polarization—and especially on interventions to address it—have been conducted in WEIRD contexts (Hartman et al., 2022; Torcal et al., 2023; though see Greene et al., 2025 for an example from Mexico). We recognize the importance of incorporating diverse perspectives to fully understand political conflict and, whenever possible, we draw upon the insights of scholars and research conducted outside the United States to enrich our understanding of partisan animosity. For example, we sought to highlight the growing body of research on affective polarization conducted outside the United States (Amitai et al., 2023; Bergman & Fernández, 2025; Boxell et al., 2024; Garzia et al., 2023; Gidron et al., 2019; Hsiao & Yu, 2025; Reiljan, 2020, Wagner, 2021) and at times draw upon research conducted by Middle Eastern scholars studying the Israel-Palestine conflict (Goldenberg et al., 2018; Halevy et al., 2022; Halperin, 2008; Halperin, Russell, Trzesniewski, et al., 2011; Waytz, Young, & Ginges, 2014). Future research on partisan animosity and its interventions should extend beyond the United States and Europe. Our citations also span contemporary research published just this year, as well as foundational work on intergroup relations and social identity conducted in the mid-twentieth century.

We do not assume that findings that come predominantly from a U.S. context will generalize to other cultural contexts. On the other hand, we also do not assume that findings from the United States apply only in that context. Surely, there are some general principles of human psychology that apply across cultures. The extent to which findings are cross-culturally generalizable is an open empirical question. With respect to blame, theories and findings with roots in U.S.-based research have often proved useful to understanding how blame operates in other cultures (for example see Chernyak et al., 2013; Clark et al., 2014; Eriksson et al., 2021; Feinberg et al., 2019; McNamara et al., 2019; Sarkissian et al., 2010).

## Conclusion

The alarming rise in partisan animosity across the globe and its pernicious consequences has motivated scientists around the world to study interventions to reduce it. In this paper, we have sought to offer a novel perspective on the issue by conceptualizing partisan animosity through the lens of blame. We draw from a rich body of literature that sheds light on how blame operates, highlighting unexplored potential mechanisms to target reducing partisan animosity. By conceptualizing partisan animosity as an instance of blame, we have introduced a unifying framework to understand current interventions and to offer ideas for future research. Our analysis underscores the importance of understanding the blame-based psychological mechanisms underlying partisan animosity. We hope our work can help scholars and practitioners in the field in the quest to reduce toxic polarization—while retaining ideological diversity—around the world.
